# Metabolomics profiling of hypervitaminosis A in South African preschoolers is characterised by modified serum lysophospholipids and oxylipins

**DOI:** 10.1017/S0007114525103656

**Published:** 2025-06-14

**Authors:** Rairaja Cohen, Jesse Sheftel, Jennifer Luevano, Meredith O. Kelly, Rob K. Fanter, Martha E. van Stuijvenberg, Muhammad A. Dhansay, Alex Brito, Sherry A. Tanumihardjo, Michael R. La Frano

**Affiliations:** 1 Department of Food Science and Nutrition, California Polytechnic State University, San Luis Obispo, CA, USA; 2 University of Wisconsin-Madison, Madison, WI, USA; 3 Cal Poly Metabolomics Service Center, California Polytechnic State University, San Luis Obispo, CA, USA; 4 Department of Chemistry and Biochemistry, California Polytechnic State University, San Luis Obispo, CA, USA; 5 College of Agriculture, Food and Environmental Sciences, California Polytechnic State University, San Luis Obispo, CA, USA; 6 Non-Communicable Diseases Research Unit, South African Medical Research Council, Cape Town, South Africa; 7 Division of Human Nutrition, Department of Global Health, Stellenbosch University, Cape Town, South Africa; 8 Department of Paediatrics and Child Health, Stellenbosch University, Cape Town, South Africa; 9 School of Nutrition and Dietetics, Faculty of Rehabilitation and Quality of Life Sciences, Universidad San Sebastián, Patagonia Campus, Puerto Montt, Chile; 10 Roy J. Carver Biotechnology Center, University of Illinois Urbana-Champaign, Urbana, IL 61801, USA; 11 Burden of Disease Research Unit, South African Medical Research Council, Cape Town, South Africa

**Keywords:** Vitamin A, Hypervitaminosis A, Lipidomics, Oxylipins, Nutritional metabolomics, Preschoolers, South Africa

## Abstract

Evidence indicates hypervitaminosis A may be attributed to overconsumption of natural preformed vitamin A (VA) and overlapping VA intervention strategies. Hypervitaminosis A can disrupt metabolic processes; however, the extent and mechanisms of these impacts are not well understood. This study aims to assess metabolic differences related to hypervitaminosis A and VA supplementation by performing metabolomics analysis. A subsample of South African preschoolers participating in the country’s VA supplementation programme was selected. Participants were divided into two groups: adequate VA (*n* 15; 0·59–0·99 µmol/g total liver reserve and high VA (*n* 15; ≥ 1·0 µmol/g total liver reserve). Serum samples were collected at baseline and 28 d after consuming a 200 000 IU VA supplement. Lipidomics and oxylipins assays were conducted using ultraperformance LC-MS. At baseline, unsaturated lysophosphatidylcholines and unsaturated phosphatidylcholines were significantly lower in the high VA group (*P* < 0·05). A group-by-time interaction with VA supplementation was observed for polyunsaturated lysophosphatidylcholines and polyunsaturated phosphatidylcholines (*P* < 0·05). Additionally, a group effect was noted for oxylipins, and a time effect in response to VA supplementation was seen with decreased arachidonic acid and lipoxygenase- and non-enzymatically derived oxylipins (*P* < 0·05). Hypervitaminosis A is associated with modifications in lipids involved in cell structure and signalling, particularly unsaturated lysophosphatidylcholines and phosphatidylcholines. Further research is needed to identify the mechanisms behind these modifications, their physiological effects and their potential as biomarkers of elevated vitamin A status.

Vitamin A (VA) is a fat-soluble vitamin available in various forms^(1)^. Pre-formed VA, primarily in the form of retinyl esters, is found in animal sources such as milk, cheese, eggs, fish and liver. Provitamin A carotenoids, including beta-carotene, *α*-carotene and beta-cryptoxanthin, are present in the pigments of orange, yellow and red fruits and vegetables. Additionally, synthetic, water-soluble forms of VA are available in some supplements^(2)^. Adequate intake of VA is essential as it plays significant roles in reproduction, growth, development, vision and immunity. During fetal development, sufficient VA is essential for proper organogenesis and lung maturation. Throughout the human lifespan, adequate vitamin A status (VAS) is crucial for growth, enabling the synthesis of VA-specific proteins and supporting cell differentiation and proliferation. VA is also critical for dim-light vision through its involvement in the rhodopsin pathway. Furthermore, VA enhances immunity by stimulating T-regulatory cells, phagocytic activity, cytokine production and natural killer cells, while inhibiting proinflammatory cells^(3)^.

Vitamin A deficiency (VAD) is a global health concern, particularly in low- and middle-income countries. It has been estimated to affect approximately 190 million children and 19 million pregnant women, contributing to high rates of morbidity and mortality among young children worldwide^(4)^. VAD can lead to decreased cell differentiation, compromising epithelial tissue, weakening the immune system and increasing the frequency and severity of infections, which raises the risk of death. VAD can also cause night blindness, growth stunting and xerophthalmia^(5)^. Additionally, a recent meta-analysis showed VAD increases the risk for neonatal respiratory distress syndrome and pneumonia^(6)^. While the consequences of VAD are well-documented, the acute and chronic symptoms of VA hypervitaminosis are also well-defined. These symptoms are often associated with high-dose VA supplementation without proper medical supervision and include liver damage, alterations in bone metabolism, increased risk of osteoporosis and bone fractures, fetal malformations and acute gastrointestinal issues^(1)^. When individuals are at risk of developing hypervitaminosis A, VA can accumulate to toxic levels. Although the risk of toxicity from consuming VA through a normal diet is uncommon, certain populations that frequently consume retinyl ester-rich preformed VA food sources may be more susceptible^(7)^. Hypervitaminosis A can negatively impact various metabolic processes; however, the full extent and mechanisms of these influences are not yet completely understood. Research is needed to assess the influence of hypervitaminosis A on metabolism and to identify potential biomarkers of elevated VAS. Complex lipids and oxylipins have been shown to be significantly modified by changes in VAS^(8,9)^ although these studies primarily focused on low VAS.

Establishing biomarkers of elevated VAS through metabolomics analysis could enhance clinical diagnostics and enable comprehensive population-wide assessments of VAS, thereby improving public health interventions. The most commonly used method for assessing VAS is measuring serum retinol concentrations. However, serum retinol is homeostatically regulated to remain within a narrow range, revealing deficiency or toxicity only when it is severe. Additionally, serum retinol levels decrease during infection and inflammation because it binds to retinol-binding protein, a negative acute-phase protein, further reducing the accuracy of this method. Retinol isotope dilution (RID) is a sensitive method as it measures total VA reserves in the liver^(10)^. While RID is accurate, it is impractical for population-wide use due to its high cost and complexity^(11)^. The aim of this study is to identify metabolic changes in complex lipids and oxylipins related to hypervitaminosis A and VA supplementation in a group of South African preschoolers, whose VAS was measured using RID.

## Participants and methods

### Ethical approval

This study was a secondary analysis of a subsample from a VA supplementation intervention study performed by Stuijvenberg *et al*.^(12)^ in Calvinia, located in the Hantam district of the Northern Cape province of South Africa. The original longitudinal cohort study adhered to the guidelines set forth in the Declaration of Helsinki. All procedures involving human participants were approved by the Ethics Committee of the South African Medical Research Council (ECO16-5/5/2015) and the Ethics Committee of the Northern Cape Department of Health (NC_2016RP47_96). The study was registered at ClinicalTrials.gov (NCT02915731). Written informed consent was obtained from the legal guardians of all preschool children (*n* 95).

### Study population

Flyers and radio messages were used to provide information about the study to parents and caregivers who would serve as points of contact for participants. Children aged 30–60 months who were brought to the site and met the inclusion criteria – generally healthy, without fever and with Hb levels ≥ 90 g/l – were included^(12)^. The sample size of the primary intervention study was determined based on expert panel recommendations for VAS assessment using the paired RID, which suggested 15–30 children per group for a supplement intervention^(11)^. In that study used to determine the sample size of the primary intervention, each participant served as their own control by having a baseline measurement before the supplement and a follow-up after the supplement was administered. The study began 5 months after the last VA supplementation campaign. Children who had received a high-dose VA supplement within the previous month were assessed at baseline but not followed up. Temperature was measured using a Braun ThermoScan PRO 6000 digital ear thermometer, and Hb levels were determined using a HemoCue instrument. Height was measured to the nearest 0·1 cm using a portable SECA 214 Leicester Height measure, and weight was measured to the nearest 0·05 kg using an electronic load cell scale, with children wearing light clothing and no shoes. Children with moderate anaemia (Hb < 90 g/l) or a body temperature > 38°C were excluded. For this secondary analysis, thirty of the ninety-five children were selected for the subsample. Fifteen children with adequate VAS (0·59 µmol/g–0·99 µmol/g total liver reserve (TLR)) and fifteen children with hypervitaminosis A (≥ 1·0 µmol/g TLR) were randomly selected. Each child acted as their own control, with blood samples collected during the RID procedure immediately before consuming a 200 000 IU VA capsule and 28 d afterwards.

### Design and field procedures

The original study was an intervention study conducted from April to June 2016, assessing total body stores (TBS) and TLR of VA using the RID technique in a cohort of preschool children before and 4 weeks after administration of a high-dose VA supplement^(12)^. Each child served as their own control. The RID assessment involved drawing blood samples from each child via venipuncture at two time points, before and after a 14-d isotopic mixing period. For each RID assessment, a baseline blood sample was collected before orally administering 1·0 µmol [14, 15]-^13^C_2_-retinyl acetate in soybean oil (183 µl) using a positive displacement pipette, followed by 1 ml of sunflower oil and a high-fat snack. The second blood sample was taken after the 14-d equilibration period. During this period, participants were instructed to consume a relatively low VA diet, specifically avoiding liver and liver products. Food intake during the mixing period was retrospectively obtained and recorded. After the second blood draw, each child received a 200 000 IU VA capsule, as recommended by the WHO for prevention of VAD^(13)^. The RID procedure was repeated 4 weeks later. All blood samples were centrifuged within 1 h of collection, and the serum was transferred to cryovials for various assays. The cryovials were stored at –20°C at the local hospital for a maximum of 5 d before being transported to the South African Medical Research Council, where they were stored at –80°C and then transported to the University of Wisconsin-Madison. Z-scores for height and weight at baseline, as well as weight at the third assessment, were calculated using the 2006 WHO growth standards^(14)^. Demographic information was obtained through an interview-administered questionnaire. The children’s birth dates and history of VA supplementation were obtained from their clinic cards. Habitual consumption of liver was assessed using a quantified liver intake frequency questionnaire.

### Diets

All children in the original intervention study consumed a diet that included regular liver intake, as well as VA-fortified bread and VA-fortified maize meal. Before both the baseline and post-supplementation blood draws, mothers were advised to feed their children breakfast while avoiding high VA foods, such as eggs and liver. During the RID procedure, after the administration of isotope-labelled soybean oil, all children were given a half slice of unfortified bread with 20 g of peanut butter and 1 ml of soybean oil. During the 14-d mixing period, participants were advised to avoid liver and liver-containing products to maintain a low VA diet until the next blood draw^(12)^.

### Measurement of total body stores, total liver reserves and serum retinol concentrations

TBS were calculated from the tracer-to-tracee ratio (TTR) using the following formula^(15,16)^:

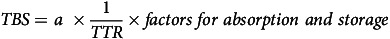

where *a* is the amount of ^13^C_2_-retinyl acetate in the dose (1·0 µmol). TLR were estimated from TBS using the following formula:

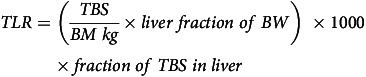

where BM is body mass (kg) and liver fraction of BW was estimated as 3 % in preschool children. It was assumed that 80 % of TBS were in the liver storage pool of children with adequate hypervitaminotic VAS. During the purification of retinol for injection into the GC-combustion ratio mass spectrometer, serum retinol concentrations were determined^(15,17)^.

### Analytical methods

Lipidomics and oxylipins assays were performed using previously published methods utilising protein precipitation extraction with ultraperformance LC-MS (UPLC-MS)^4^. As described by Johnson *et al.*, 2022^(9)^, to begin the assays, 25 ul of plasma was added to 1·5 ml tubes along with 10 ul of surrogate internal standard solution (containing arachidonic acid-d8 and ceramide C17) and 750 ul chilled methanol. Each sample was vortexed for 30 s and then centrifuged at 15 000 ***g*** for 10 min. The acquired supernatant was transferred to 1·5 ml amber glass HPLC vials. Centrifugal vacuum evaporation was used to dry the vials before reconstituting them in 100 ul 3:1 acetonitrile: methanol solution containing the instrument internal standards’ 1-cyclohexyl ureido, 3-dodecanoic acid and 1-phenyl 3-hexadecanoic acid urea at 100 nM. The reconstituted solution was vortexed for 30 s before being placed on ice for 10 min. After icing, the solution was transferred to microfilter tubes and then centrifuged at 10 000 ***g*** for 3 min. The acquired supernatant was transferred to an amber glass HPLC vial for analysis using the UPLC-MS. The UPLC-MS analysis was conducted on a Waters Acquity I-Class UPLC (Waters) coupled with an API 4000 QTRAP (Sciex) quantified with AB Sciex MultiQuant version 3.0. The lipidomics assay was performed using a 150 × 3·0 mm, 3µm Prosphere HP C4 column (Grace) with a gradient that had mobile phases consisting of solvent A as 95:5:0·1 v/v/v 10 mM ammonium acetate and solvent B as 99·9:0·1 v/v methanol/acetic acid^(9)^. The method screened for phospholipids, sphingolipids and ceramides utilising positive mode electrospray ionisation with multiple reaction monitoring^(18)^. The oxylipins assay was performed using a 100 × 2·1 mm, 1·7 μm Waters BEH C18 column with a gradient that had mobile phases consisting of solvent A as 99·9:0·1 v/v water/ acetic acid and solvent B as 90:10 v/v acetonitrile/isopropanol^(9)^. The oxylipins method screened for oxylipins, PUFA and nitro-lipids through the use of negative mode electrospray ionisation with multiple reaction monitoring^(19)^. Data are in peak area, with lipidomics and oxylipin assay metabolites normalised to the internal standards’ ceramide C17 and arachidonic acid-d8, respectively. For reasons of quality control, compounds whose background (as determined by method blank response) was greater than 50 % of the average sample response were excluded from the final dataset. To assess reproducibility, three replicates of the present study samples were separately processed and analysed. A pooled plasma sample from a different study was used as a long-term reference Quality control (QC) sample for an inter-study assessment. All samples were randomised and run in a single batch for each assay.

### Statistical analysis and justification of sample size

Differences in participant characteristics, such as weight, height and age, between the adequate and high VAS groups were tested using independent *t* tests on raw values. Similarly, differences in liver VA intake, serum retinol, TLR and TBS were measured using the same method (Table 1). To examine metabolite differences between the adequate and high VAS groups at baseline, a mixed model was fitted with random effects for participant ID and fixed effects for weight, height, sex/gender and age. Post-supplementation changes in VA assessment measures, including serum retinol, TLR and TBS, were assessed using a general linear model. To analyse the post-supplementation influence on metabolites over time between all groups, a general linear model was used with random effects for participant ID and fixed effects for weight, height, sex/gender and age. Prior to all statistical analyses, data were assessed using the Shapiro–Wilk test. Normally distributed data were analysed in their raw form, while non-normally distributed data were log-transformed. Results were adjusted for multiple comparisons of 102 lipidomics assay metabolites and 30 oxylipin assay metabolites using the Benjamini–Hochberg procedure at a false discovery rate (FDR, q) of 0·05^(20)^. Before performing partial least squares-discriminant analysis, data were covariate-adjusted for weight, height and age using a linear model to describe differences in metabolite values across all groups, utilising the RStudio 4.0.3 environment^(21)^. For multivariate analysis, data were auto-scaled. VAS was used as the classifier, and models were validated using 10-fold cross-validation. Partial least squares-discriminant analysis was performed in MetaboAnalyst 4.0^(21)^. Chemical similarity enrichment analysis was performed using ChemRICH to compare adequate *v.* high VAS groups at baseline. This enrichment approach is based on chemical ontologies and structural similarity, addressing the lack of complex lipids and oxylipins in pathway enrichment software that relies on the biological pathway commonality of metabolites^(22)^. G * Power: Statistical Power Analyses were utilised to ascertain the sample size for the secondary analysis using metabolomics with a statistical significance of an *α* = 0·05 and a power of 0·95 (G * Power, version 3.1.9.7 for Windows)^(23)^. This determination was based on a previous experiment conducted by our lab, which analysed differences in complex lipid and oxylipin levels between women with varying deficient *v.* adequate VAS based on serum retinol^(9)^. It was determined that a sample size of five participants was needed. Due to this present study being conducted in children with elevated *v.* adequate liver VAS but similar serum retinols, triple this amount was used for a sample size of fifteen participants per group.

**Table 1.** Baseline characteristics of study participants (Mean values and standard deviations)

Characteristic	Adequate VA group (*n* 15)		High VA group (*n* 15)		P^*^
	Mean	sd	Mean	sd	
Age (month)	49·2	7·0	46·0	9·9	0·20
Weight (kg)	14·4	2·1	13·1	2·1	0·10
Height (cm)	97·3	4·7	94·0	6·5	0·10
Sex/gender (% female)	50	50	60	60	–
Serum-based TBS (µmol)	318·1	68·6	845·5	194·2	<0·001
Serum-based TLR (µmol/g)	0·6	0·1	1·8	0·5	<0·001
Baseline serum retinol (µmol/l)	1·1	0·3	1·1	0·4	1·00
Liver VA intake (µg retinol activity equivalents/d)	109·9	149·4	656·8	703·6	<0·004

TBS, total body stores; TLR, total liver reserves; VA, vitamin A.

* Two-sided *t* test: level of significance *α* = 0·05.

## Results

### Participant characteristics

TLR and TBS were significantly higher in the hypervitaminotic VA group compared with the adequate VAS group at baseline (*P* < 0·001) (Table 1). There were no significant differences in serum retinol concentrations, age, height or weight. Habitual VA intake from liver (retinol activity equivalents) was significantly higher in the hypervitaminotic VA group *v.* adequate VA group (*P* < 0·004) (Table 2). Post-VA supplementation, TLR and TBS significantly increased in the hypervitaminotic VA group *v.* adequate VA group at baseline (*P* < 0·02) (Table 3). Serum retinol did not change (Table 3).

**Table 2.** Baseline comparison of metabolites between adequate and high vitamin A groups (Mean values and standard deviations)

Metabolite name	Metabolite class	Adequate VA group (*n* 15)		High VA group (*n* 15)			
		Mean	sd	Mean	sd	Fold change	P^*^
LPC 16:1	Lysophosphatidylcholine	141041·0	44423·5	93036·8	41603·5	0·7	0·003
LPC 18:1	Lysophosphatidylcholine	1531988·7	726156·8	1140960·8	465006·8	0·7	0·04
LPC 20:3	Lysophosphatidylcholine	203360·6	124803·6	119291·9	46751·7	0·6	0·02
LPC 20:4	Lysophosphatidylcholine	466457·6	232583·8	347895·0	129521·3	0·7	0·02
LPC 22:4	Lysophosphatidylcholine	18556·8	9612·1	11932·2	5009·2	0·6	0·02
LPC 22:5	Lysophosphatidylcholine	56452·7	30790·1	39371·7	22011·5	0·7	0·003
LPE 18:1	Lysophosphatidylethanolamine	7018·2	3142·6	4802·7	4061·6	0·7	0·02
PC 32:1	Phosphatidylcholine	3042833·2	800294·6	2466962·2	628328·1	0·7	0·03
PC 34:3	Phosphatidylcholine	904774·4	206708·2	669745·2	220625·2	0·7	0·002
PC 34:4	Phosphatidylcholine	63678·9	26731·8	45015·0	21207·8	0·7	0·03
PC o-40:0	1-O-alkyl-phosphatidylcholine plasmalogen	146140·6	33308·0	117797·9	36076·8	0·6	0·02
SM 18:1 20:1	Sphingomyelin	616154·1	142513·1	510611·6	127111·0	0·6	0·04
*α*-Linolenic acid	*n*-3 fatty acid	4772·0	3188·4	2751·6	2046·9	0·6	0·03

LPC, lysophosphatidylcholine; LPE, lysophosphatidylethanolamine; PC, phosphatidylcholine; SM, sphingomyelin.

Metabolomics data are in the peak area.

Tests: fold-change, High VA /Adequate VA.

* Two-sided *t* test: level of significance *α* = 0.

**Table 3.** Vitamin A concentrations across groups (Mean values and standard deviations)

Variable	Adequate VA group (*n* 15)				High VA group (*n* 15)				P^*^
	Baseline		Post-supplementation		Baseline		Post-supplementation		
	Mean	sd	Mean	sd	Mean	sd	Mean	sd	
Serum-based TBS (µmol)	318·1	68·6	434·6	125·3	845·5	194·2	836·3	212·0	0·01
Serum-based TLR (µmol/g)	0·6	0·1	0·8	0·2	1·8	0·5	1·8	0·6	0·02
Serum retinol (µmol/l)	1·1	0·3	1·2	0·4	1·1	0·4	1·1	0·4	0·70

TBS, total body stores; TLR, total liver reserves.

* Mixed model: level of significance *α* = 0.

### Metabolomics

A total of 102 lipidomics assay and 30 oxylipin assay metabolites were detected. At baseline, metabolites lower in the high VA group included three unsaturated phosphatidylcholines (PC), one saturated plasmalogen PC, six unsaturated lysophosphatidylcholines (LPC), one unsaturated lysophosphatidylethanolamine, one sphingomyelin and one *n*-6 fatty acid (*P* < 0·04) (Table 2, online Supplementary Table 1). After FDR adjustment, none of the specific metabolites was found to be significantly different (FDR *P* < 0·05).

**Fig. 1.**
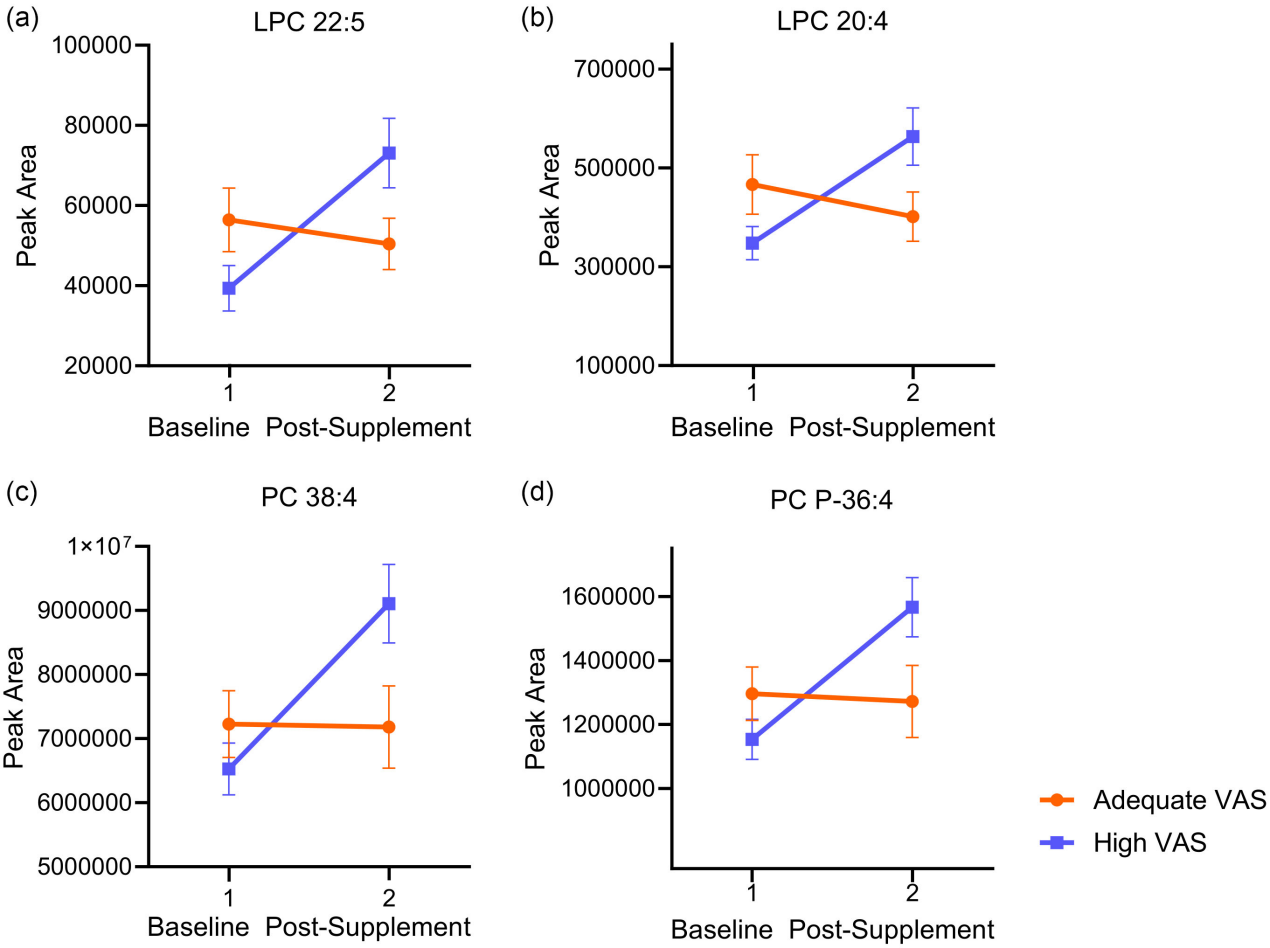
Changes in metabolites before and after vitamin A supplementation. (a) LPC 22:5, (b) LPC 20:4, (c) PC 38:4, (d) PC P-36:4. LPC, lysophosphatidylcholine; PC, phosphatidylcholine; VAS, vitamin A status.

**Fig. 2.**
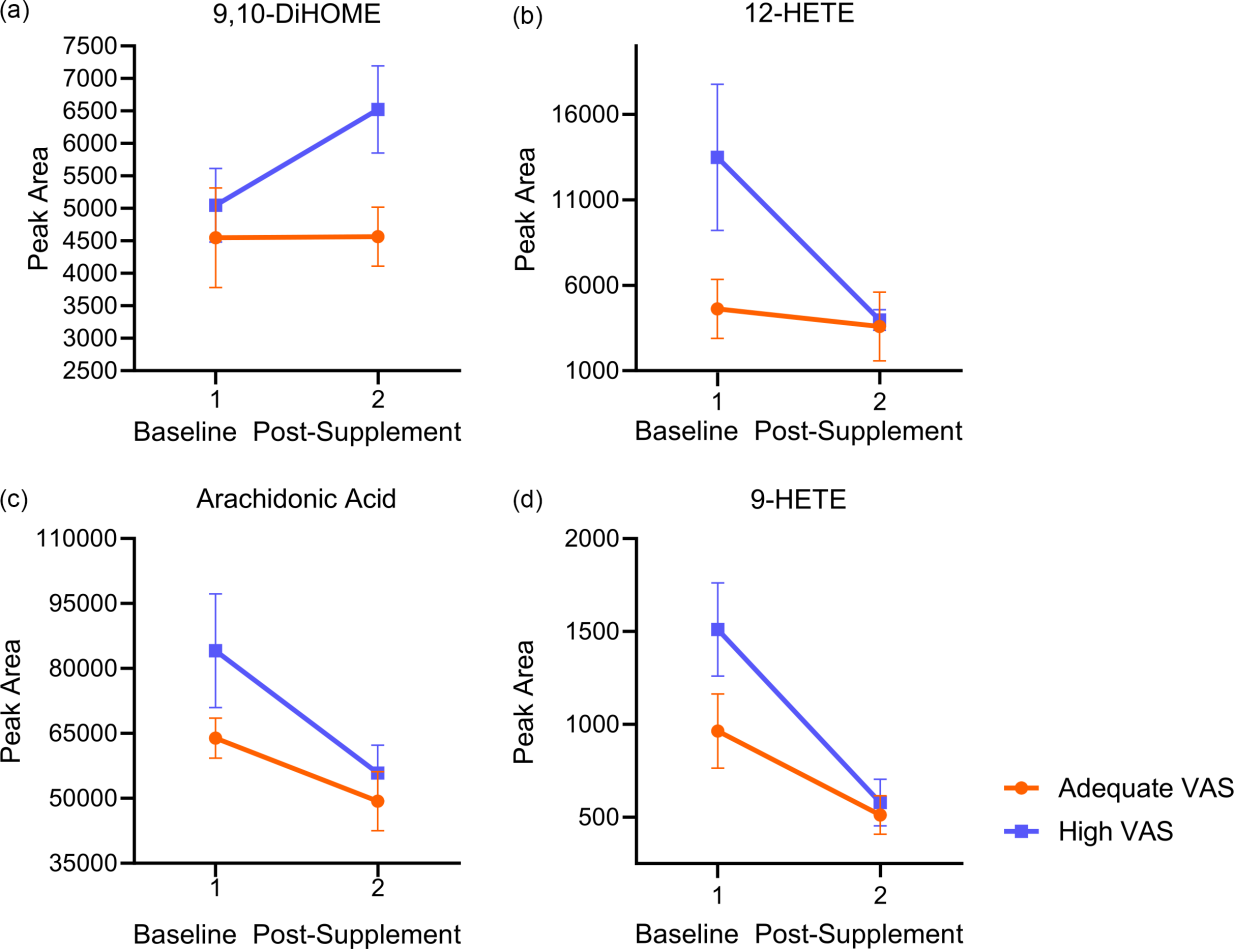
Changes in metabolites before and after vitamin A supplementation. (a) 9,10-DiHOME, (b) 12-HETE, (c) arachidonic acid, (d) 9-HETE. VAS, vitamin A status.

Post-supplementation, a group-by-time effect exhibited a differential response in the adequate VA *v.* hypervitaminotic VA group, with lipids decreasing or increasing, respectively (Table 4, online Supplementary Table 2). Modified lipid metabolites included six polyunsaturated PC, one saturated PC, five polyunsaturated plasmalogen PC, two polyunsaturated phosphatidylethanolamine plasmalogens, one saturated phosphatidylethanolamine and 9,10-EpODE (*P* < 0·05) (Figure 1, Table 4). After FDR adjustment, no individual metabolites were found significant (FDR *P* > 0·05). A complete summary of the group-by-time effect metabolite results is available in online Supplementary Table 2. There was a group effect for 9,10-DiHOME and 12-HETE, with both being higher in the high VA group (*P* < 0·05) (Figure 2(a)–(b), online Supplementary Table 2). A time effect was observed in both groups, with various reductions post-supplementation in arachidonic acid and four of its lipoxygenase-derived oxylipins, such as 12-HETE, 11-HETE, LTB4 and 6-*trans*-LTB4, along with non-enzymatically-derived 9-HETE (*P* < 0·05). Cytochrome P450-derived 9,10-EpOME and 11,12-EpETrE also decreased (*P* < 0·05) (Figure 2(b)–(d), online Supplementary Table 2). A time effect was also observed, with elevations in three polyunsaturated PC, one saturated PC and five polyunsaturated plasmalogen PC (*P* < 0·05). Principal component analysis (PCA) did not display group differences (online Supplemental Figure 1.). The partial least squares-discriminant analysis model did not differentiate groups along either component one or component two, and the model was not predictive, with low R2 and Q2 values from cross-validation (online Supplemental Figure 2). As determined using ChemRICH software, chemical similarity enrichment analysis comparing adequate *v.* high VAS groups at baseline revealed unsaturated PC and unsaturated LPC as significantly impacted metabolite clusters (*P* < 0·05, FDR *P* < 0·05) (online Supplementary Table 3).

**Table 4.** Group-by-time interaction results pre- and post-supplementation for adequate *v.* high vitamin A groups (Mean values and standard deviations)

Metabolite name	Adequate VA group (*n* 15)				High VA group (*n* 15)				P^*^
	Baseline		Post-supplementation		Baseline		Post-supplementation		
	Mean	sd	Mean	sd	Mean	sd	Mean	sd	
LPC 18:2	2522255	920851·2	2085065	688218·7	1951173	707727·3	3000127	1547834	0·023
LPC 20:3	203360·6	124803·6	157647·7	66817·5	119291·8	46751·7	211820·3	103056·7	0·008
LPC 20:4	466457·5	232583·8	401661·1	194279·8	347895	129521·3	563648·9	224698·2	0·010
LPC 22:4	18556·8	9612·0	15629·7	7946·3	11932·2	5009·24	23302·7	11324·8	0·004
LPC 22:5	56452·74	30790·1	50416	24801·1	39371·7	22011·5	73078·2	33555·7	0·002
LPE 18:1	7018·1	3142·2	6152·8	3849·7	4802·7	4061·6	9698·6	8876·0	0·041
PC 34:4	63678·9	26731·8	57118·3	28349·3	45015·0	21207·8	74177·1	36708·4	0·017
PC 38:0	518675·2	172717·4	429535·7	192922·6	415042·4	124059	524260·9	145019·1	0·022
PC 38:3	3751016	1419766	3612143	1464675	2916639	849155·5	4393636	1838421	0·038
PC 38:4	7228902	2031120	7179523	2493298	6526654	1571204	9106957	2486113	0·029
PC 38:5	2613689	594295·4	2712513	857976	2314761	583957·7	3487533	989748·3	0·013
PC o-34:3	889361	262984·1	884251	413224·1	677252·7	219583·5	933603·5	300958·1	0·046
PC o-36:5	1101481	294662·2	1100307	366028·3	975060·5	220814·1	1333793	335462·7	0·039
PC o-38:5	1526168	366814·2	1508477	557694·3	1357725	365024·2	1837396	453042·5	0·042
PC p-36:4	1296247	322751·5	1272016	436822·4	1153659	242207·4	1566255	359255·8	0·023
PC p-38:5	677169·8	141204·4	669632·6	210939·5	620252·1	178435·7	817692·1	234828	0·033
PE 38:0	4046·6	2394·2	3240·8	1699·3	3529·7	1488·1	5580·6	1416·2	0·003
PE p-38:4	13567·1	5357·22	9924·9	4081·9	10361·5	3701·1	11675·8	3668·0	0·031
PE p-38:5	8935·5	3627·4	8371·5	4154·7	8805·0	3554·3	12454·0	5446·1	0·048
9,10-EpODE	1179·9	515·7	984·4	638·2	1116·4	935·3	1646·1	1558·3	0·027

LPC, lysophosphatidylcholine; LPE, lysophosphatidylethanolamine; PC, phosphatidylcholine; SM, sphingomyelin.

Metabolomics data are in the peak area.

* Mixed model: level of significance *α* = 0·05.

## Discussion and Conclusions

This study aimed to identify metabolic changes in South African preschool children with adequate and high VAS in response to a VA supplementation programme. Our observational study was designed as a hypothesis-generating investigation rather than a hypothesis-driven study. Serum samples were analysed using lipidomics and oxylipin assays via UPLC-MS. At baseline, numerous unsaturated LPC and unsaturated PC were found to be modified in children with high VAS. Post-VA supplementation, a differential response was observed between children with adequate and high VAS. Additionally, group and time effects were noted in various oxylipins.

Metabolomic analyses can be used to further understand the influence of VAS on metabolism. As highlighted in a recent 2024 study, metabolomics is a useful tool to identify adverse outcome pathways in nutrient deficiencies and toxicities. This study proposed remodelled lipids as potential markers of toxicity^(24)^. To our knowledge, the first metabolomic characterisation of VA was done in 2019 by Zhong *et al*.^(25)^. This study focused on the effect of non-alcoholic fatty liver disease on VA metabolites, analysing retinol, retinyl palmitate and multiple retinoic acids and established that the VA metabolome is significantly modified in non-alcoholic fatty liver disease^(25)^. A recent non-targeted metabolomics study identified VA as a significantly upregulated metabolite in non-alcoholic fatty liver disease, alongside altered bile acid metabolism, and proposed VA as a potential biomarker for hepatic metabolic dysfunction^(26)^. The first metabolomic characterisation of VAD was performed by La Frano *et al.* using a rodent model^(8)^. This study utilised metabolomics (primary and secondary metabolites), lipidomics and lipid mediator assays to assess metabolic changes in rodents with adequate VAS *v.* VAD. It was revealed that VAD resulted in lower hepatic concentrations of primary bile acids and cell membrane lipids associated with the Kennedy pathway, including phospholipids, ceramides and sphingomyelins^(8)^. Additionally, levels of metabolites known to be influenced by gut microbiota, such as ursodeoxycholic acid and trimethylamine N-oxide (TMAO), were decreased with VAD. A follow-up metabolomics characterisation of VAD was conducted in 2022 by Johnson *et al.* on lactating women in the Philippines^(9)^. This study also found decreased concentrations of multiple lipids, including several sphingomyelins, ceramides, lysophospholipids (LPL) and phospholipids, in women with VAD. In addition to providing further evidence for decreased cell membrane lipid levels with VAD, reductions in many unsaturated fatty acid derivatives, including *n*-6 and *n*-3 oxylipins, and endocannabinoid-like N-acylethanolamides such as linolenoyl ethanolamide (LEA) and palmitoleoyl ethanolamide, were observed. Both studies provided evidence that lipid metabolism is most disrupted with VAD.

In the present study, numerous phospholipids and LPL were found to be lower in children with hypervitaminosis A at baseline. A differential response was observed post-VA supplementation between those with adequate and high VAS. Phospholipids are a class of lipids composed of a polar, phosphate-containing headgroup attached to non-polar hydrocarbon chains. They are a major component of cell membranes, contributing to membrane permeability and facilitating the transport of lipids, fat-soluble vitamins and hormones. Phospholipids also provide structural support for many catalytic processes, play a role in signal transduction and cell signalling and are present in all major tissues^(27,28)^. Post-VA supplementation, an increase in plasmalogen phospholipids was observed in children with high VAS. Plasmalogens are a subclass of phospholipids characterised by an alkyl chain bound to the sn-1 position of the glycerol backbone via a vinyl–ether bond, rather than an acyl chain bound by an ester bond. The vinyl–ether bond is more hydrophobic and prone to oxidation, which results in increased lipid packing and membrane thickness and decreased membrane fluidity. Plasmalogens also act as scavengers for radical species and play a major role in signal transduction^(29)^. Although data from similar studies on high VAS are lacking, plasmalogen levels have been shown to be modified in various disease states and^(29–32)^ are suggestive of lipid remodelling^(33,34)^.

With respect to the lower phospholipids observed in baseline samples of children with hypervitaminosis A, there is currently no evidence directly associating hypervitaminosis A with these lipid changes. Some research studies have linked phospholipid levels with VAD, showing decreased liver phospholipid content potentially resulting from the downregulation of acetyl-CoA carboxylase due to VAD, as well as a general reduction in circulating lipid mediators^(8,9)^. Research also exists concerning the effect of VA administration on phospholipid levels. All-*trans* retinoic acid, the major bioactive VA metabolite, has been shown to activate retinoid X receptors that deplete PC-bound PUFA, mainly PC-bound 18:2, to activate the serine/threonine-specific protein kinase Akt. Evidence also shows that all-*trans* retinoic acid elevates levels of phosphotidylinosital-3-kinase by controlling expression, stability and phosphorylation of key enzymes in its metabolic pathway^(35)^. The daily administration of VA (15 mg of VA acetate) for 6 months in geriatric participants elevated serum phospholipids but declined to their initial levels 3 months after discontinuing administration^(36)^. A second human study that included receipt of VA daily (100 000 IU) for 36 months also observed the same elevated response during treatment and return to baseline levels upon discontinuation. The present observed levels of specific species of PC increased post-supplementation in only the high VA group. Because the baseline VAS in the VA administration studies previously described was unknown, it cannot be determined if this is only observed in those with high VAS. Additionally, because this study did not include follow-up analysis beyond 3 months, it remains unclear whether the observed elevations would decline substantially over time after supplementation. However, it is important to note that in South Africa, VA supplementation is given every 6 months to children up to the age of 5 years.

Phospholipids can be metabolised by the removal of one acyl group to produce LPL that can carry either an alkyl or an acyl chain. LPL are important signalling molecules that also play important structural roles in cells and organisms. They may also play a role in cell differentiation and immune response^(37)^. In various disease states, including cancer, the cell signalling properties of LPL have indicated potential roles in cell proliferation, survival and angiogenesis^(38)^. Decreased LPC concentrations have been observed in inflammatory processes of malignant diseases, potentially resulting from increased LPL consumption due to rapid phospholipid turnover^(39)^. LPL changes seen in this study may potentially be related to the observed PC as LPC are a breakdown product of phospholipids. Previous research has established an association between VA and products of PC. VAD has been associated with increased levels of phosphatidic acid, a breakdown product of PC, in rats^(40)^ and thus may be associated with increased PC breakdown during VAD. Phosphatidic acid is also an essential substrate for enzymes crucial to glycerophospholipid and TAG synthesis, indicating that lipid remodelling may be occurring during VAD^(41)^. VAD has also been associated with a decrease in sphingomyelin and LPC, in rat hearts and a significant decrease in total phospholipid content in the mitochondria of rats^(40,42)^. Past studies have shown an association between LPL and VAS, specifically with serum retinol status^(40)^. Additionally, VAD anaemia has recently been proposed as a distinct entity from iron deficiency anaemia. LPC as well as PUFA have been proposed as potential biomarkers for VAD anaemia^(43)^. Our study provided evidence supporting the idea that VAS affects LPL levels. Specific species of LPC levels were lower in the hypervitaminotic VAS group baseline. Post-supplementation, LPC levels in the hypervitaminotic VAS group increased. The accompanying similar changes in unsaturated LPC and PC may suggest they are associated with each other in their metabolic response to supplementation.

In the present study, there was a group effect for multiple oxylipins, and VA supplementation resulted in a time effect reduction in both arachidonic acid and many of its lipoxygenase-derived oxylipins. This suggests an VAS independent change in these lipid mediators when VAS ranges from adequate to high. Oxylipins, known to be modified in numerous disease states, are lipid mediators involved in controlling inflammation, vascular tone, coagulation and immunity^(9)^. It has been suggested that altered oxylipin profiles are mechanistic drivers for increased risk of neonatal respiratory distress syndrome and pneumonia associated with a VA status outside of the normal range, such as VAD^(6)^. It is also known that VA is capable of regulating lipid homeostasis and influencing the expression of genes involved in fatty acid metabolism^(44)^. As derivatives of PUFA often cleaved from PL by phospholipase A2 (PLA2), many studies observe oxylipins to be modified in concert with PL. Thus, an altered VA status could lead to dysregulation of these pathways, potentially affecting the availability of polyunsaturated fatty acid precursors for oxylipin synthesis, as observed here in the case of high VA status. As also previously observed with VAD, this high VAS can result in an altered oxylipin profile that may be linked to changes in either the previously mentioned PLA2 or the activity of enzymes responsible for oxylipin production, such as cyclooxygenases, lipoxygenases and cytochrome P450 enzymes^(45)^. These enzymatic changes could result in shifts in the balance between proinflammatory and anti-inflammatory oxylipins, potentially contributing to an altered overall metabolic and inflammatory state associated with altered VA status. The change in response to supplementation and the lack of difference at baseline suggest a temporary modification in concentrations that returns over time. Furthermore, the reduction of these oxylipins along with their precursor arachidonic acid may indicate a general decline in its precursor rather than strictly enzymatic activity. This is supported by the fact that other PUFA detected, including *α*-linolenic acid, DHA and EPA acid, and their respective oxylipin derivatives, were not modified by supplementation. Although the study by Johnson *et al.* was conducted in a different geographical location, age group and VAS range, it is interesting to note that specific LPL and phospholipids lower in the VAD participants were different from those observed in the high VA group of this present study. Additionally, the VAD participants in the Johnson *et al.* study had lower oxylipins, while oxylipins in our study were not different at baseline in the high VA group^(9)^. This suggests that the complementary assessment of LPC, PC and oxylipins may provide insight into assessing a wide range of VAS. However, a larger study collecting quantitative lipidomic and oxylipin data with a more diverse population and encompassing the range of VAS is required to confirm these findings.

The WHO recommends intermittent high-dose VA supplementation to children aged 6–59 months in countries with a high risk of VAD^(13)^. South Africa has been targeted for interventions against VAD based on three national surveys conducted in 1994, 2005 and 2012, which assessed serum retinol concentrations in preschool-aged children. These surveys consistently showed high rates of VAD, with a significant increase in low serum retinol levels between 1994 and 2005. South Africa is a geographically, culturally and socio-economically diverse country, with many lower-income agricultural areas where sheep liver, an excellent source of VA, is regularly consumed^(46)^. It is important to note that due to this diversity, the surveys may not provide an accurate picture of VAS across all regions, and VAD may not be a problem in some areas where socio-economic indicators suggest otherwise^(12)^. In South Africa, the National Vitamin A Supplementation Program targets children aged 6–59 months at public health facilities, providing a high-dose VA supplement every 6 months. The National Food Fortification Program mandates the fortification of wheat flour and maize meal, two staple grains in the region, with preformed VA^(46)^. VA is a fat-soluble vitamin that accumulates in the liver and other fatty tissues when consumption exceeds requirements^(7)^. Despite the potential high consumption of liver in some areas, these supplementation and fortification programmes continue to exist. Due to these overlapping interventions, the consumption of foods naturally high in VA and the lack of accurate and affordable methods for assessing VAS, there is a potential for hypervitaminosis A in South African children. The metabolomic profile of elevated VA presented in the present study contributes to a better understanding of metabolic modifications due to overlapping interventions and enhances our knowledge of hypervitaminosis A.

A strength of this study was the use of multiple lipid assays, which covered both abundant and low-concentration metabolites. The use of RID to determine VAS in each group is a notable strength, as it is considered the most accurate method for assessing VAS. The similarity in age, weight and height between the VA adequate and elevated VA groups enabled a more precise assessment of the specific population’s response to VAS. The collection of data at two time points further strengthened the study by allowing for the observation of changes over time within each participant. The small sample size (*n* 30) is a limitation that may have reduced the ability to detect more differences in lipids and oxylipins associated with VAS between groups. It also limits the statistical power to detect significant differences in metabolomic profiles between groups, which may lead to Type II errors. This limitation is particularly relevant when considering potential confounders that were not accounted for including dietary variations, genetic factors and environmental influences. While we acknowledge that the sample size is a limitation, the power calculations conducted enabled us to detect meaningful differences. Despite potential concerns regarding the sample size, our statistical analysis was robust. However, a larger sample size would strengthen our findings. Moreover, this study is limited in terms of representativeness because it was conducted in a subsample from a larger cohort, as the chosen participants may not fully represent the broader population of South African preschoolers. Both these limitations restrict the generalisability of the findings to other populations. While the collection of data at more than one time point strengthened the study, additional time points could have been included to observe short-term *v.* long-term effects. Future studies should aim to quantify these metabolites so that concentrations can be compared with other data collected in the future. Although the targeted metabolic approach used in this study allowed for the capture of a large number of lipids, an untargeted metabolomics approach could have captured polar metabolites as well, enabling the detection of more potential biomarkers.

Hypervitaminosis A in South African preschoolers is associated with modifications in numerous lipids involved in cell structure and signalling, particularly unsaturated LPC and PC. These findings provide valuable insights into the metabolic changes associated with hypervitaminosis A and may help in assessing elevated VAS. However, this study is observational, which inherently limits the ability to establish causation. Therefore, mechanistic studies are necessary to explore the underlying processes that may contribute to the observed outcomes. Further research is needed to validate the novel findings of this study related to unsaturated LPC and PC, to understand how elevated VAS modifies these lipids, to determine the resulting physiological effects and to evaluate the identified metabolites as potential biomarkers of elevated VAS.

## Supporting information

Cohen et al. supplementary material Cohen et al. supplementary material

## Supplementary material

For supplementary material/s referred to in this article, please visit https://doi.org/10.1017/S0007114525103656.
