# Supplementary material for: Metabolomics profiling of hypervitaminosis A in South African preschoolers is characterised by modified serum lysophospholipids and oxylipins
Source: Br J Nutr. 2025 Jun 17;133(11):1385–94. doi: 10.1017/S0007114525103656 (PMC12303720; doi:10.1017/S0007114525103656)

**Supplementary Table 1.** Univariate analysis comparing adequate versus high vitamin A status groups at baseline

| **Metabolite Name** | **Metabolite Class** | **Mean** | **Std Dev (Adequate VA)** | **Mean (High VA)** | **Std Dev (High, VA)** | **Fold Change** | **p-value** |
| --- | --- | --- | --- | --- | --- | --- | --- |
|  |  | **(Adequate VA)** |  |  |  |  |  |
| PC_34_3 | Phosphatidylcholine | 904774.4 | 206708.2 | 669745.2 | 220625.2 | 0.740234 | 0.002176 |
| LPC_16_1 | Lysophosphatidylcholine | 141041 | 44423.52 | 93036.84 | 41603.5 | 0.659644 | 0.002589 |
| LPC_22_5 | Lysophosphatidylcholine | 56452.74 | 30790.11 | 39371.7 | 22011.53 | 0.697428 | 0.003358 |
| LPE_18_1 | Lysophosphatidylethanolamine | 7018.156 | 3142.17 | 4802.661 | 4061.638 | 0.68432 | 0.015261 |
| LPC_20_3 | Lysophosphatidylcholine | 203360.6 | 124803.6 | 119291.8 | 46751.66 | 0.586603 | 0.016631 |
| LPC_22_4 | Lysophosphatidylcholine | 18556.84 | 9612.048 | 11932.2 | 5009.154 | 0.643008 | 0.017426 |
| PC_O_40_0_ | 1-O-alkyl-phosphatidylcholine plasmalogen | 146140.6 | 33308.01 | 117797.9 | 36076.77 | 0.806058 | 0.018375 |
| LPC_20_4 | Lysophosphatidylcholine | 466457.5 | 232583.8 | 347895 | 129521.3 | 0.745824 | 0.024096 |
| PC_32_1 | Phosphatidylcholine | 3042833 | 800294.6 | 2466962 | 628328.1 | 0.810745 | 0.025799 |
| PC_34_4 | Phosphatidylcholine | 63678.89 | 26731.81 | 45015.01 | 21207.8 | 0.706906 | 0.026929 |
| ALA__screen_ | Omega-3 fatty acid | 4772.035 | 3188.372 | 2751.587 | 2046.865 | 0.576607 | 0.030723 |
| LPC_18_1 | Lysophosphatidylcholine | 1531989 | 726156.8 | 1140961 | 465006.8 | 0.744758 | 0.03736 |
| SM_18_1_20_1 | Sphingomyelin | 616154.1 | 142513.1 | 510611.6 | 127111 | 0.828708 | 0.039165 |
| LPC_18_2 | Lysophosphatidylcholine | 2522255 | 920851.2 | 1951173 | 707727.3 | 0.773583 | 0.052949 |
| 9_10__EpODE | EpODE | 1179.861 | 515.6705 | 902.2802 | 448.6843 | 0.764734 | 0.05322 |
| PC_40_7 | Phosphatidylcholine | 141967.1 | 40582.99 | 113682.8 | 40709.93 | 0.800769 | 0.054171 |
| LPC_22_6 | Lysophosphatidylcholine | 155645.6 | 98786.72 | 107962.8 | 54339.55 | 0.693645 | 0.059776 |
| Resolvin_D2 | Resolvin | 6501.571 | 4898.782 | 3571.951 | 2367.579 | 0.549398 | 0.061435 |
| 12_HETE | HETE | 4610.538 | 6690.156 | 9976.871 | 9790.242 | 2.163928 | 0.068454 |
| PC_P_34_2_ | 1-O-alkyl-1'-enyl-phosphatidylcholine plasmalogen | 1123057 | 297505.6 | 872541.8 | 260161 | 0.776934 | 0.072108 |
| Protectin_DX | Protectin | 9854.093 | 5235.219 | 15357.32 | 11413.49 | 1.558471 | 0.073343 |
| 10_Nitrooleate | Nitro lipid | 1496.608 | 1102.419 | 1013.966 | 676.801 | 0.677509 | 0.08119 |
| PE_P_38_4_ | 1-O-alkyl-1'-enyl-phosphatidylethanolamine plasmalogen | 13567.13 | 5357.209 | 10361.52 | 3701.125 | 0.763722 | 0.083054 |
| PC_O_34_3_ | 1-O-alkyl-phosphatidylcholine plasmalogen | 889361 | 262984.1 | 677252.7 | 219583.5 | 0.761505 | 0.084106 |
| PC_30_1 | Phosphatidylcholine | 6820271 | 2014381 | 5765449 | 1265298 | 0.84534 | 0.084557 |
| PC_38_5 | Phosphatidylcholine | 2613689 | 594295.4 | 2314761 | 583957.7 | 0.88563 | 0.086496 |
| PC_32_0 | Phosphatidylcholine | 2246300 | 561407 | 1937715 | 466596.3 | 0.862625 | 0.087547 |
| SM_18_1_16_0 | Sphingomyelin | 10283404 | 2737443 | 8906080 | 1865549 | 0.866063 | 0.093561 |
| PC_38_0 | Phosphatidylcholine | 518675.2 | 172717.4 | 415042.4 | 124059 | 0.800197 | 0.10765 |
| PC_36_3 | Phosphatidylcholine | 8549163 | 2628956 | 6980137 | 1562662 | 0.81647 | 0.107909 |
| SM_d18_0_16_0 | Sphingomyelin | 991125.3 | 273337.8 | 868798.1 | 177631.4 | 0.876577 | 0.109012 |
| SM_d18_0_18_0 | Sphingomyelin | 414170.9 | 121661.3 | 348043 | 107417.6 | 0.840337 | 0.109357 |
| LTB5 | Leukotriene | 1426.138 | 483.045 | 1911.6 | 1000.275 | 1.340403 | 0.115155 |
| PC_34_1 | Phosphatidylcholine | 30386412 | 8215054 | 26357821 | 6887338 | 0.867421 | 0.118106 |
| 20_HETE | HETE | 5282.629 | 3018.758 | 9423.322 | 11371.23 | 1.783832 | 0.122735 |
| PC_34_2 | Phosphatidylcholine | 37984628 | 10983284 | 33106802 | 7486919 | 0.871584 | 0.12349 |
| PC_38_3 | Phosphatidylcholine | 3751016 | 1419766 | 2916639 | 849155.5 | 0.77756 | 0.124266 |
| 12_13_DiHOME | DiHOME | 1249.78 | 909.7836 | 868.4091 | 416.9439 | 0.694849 | 0.127049 |
| LPE_18_2 | Lysophosphatidylethanolamine | 10340.07 | 5778.329 | 7955.349 | 3721.424 | 0.769371 | 0.128303 |
| PC_36_4 | Phosphatidylcholine | 9867568 | 2525176 | 9083099 | 1947708 | 0.9205 | 0.129589 |
| PC_32_2 | Phosphatidylcholine | 689729.4 | 182086.8 | 591968.9 | 151183.1 | 0.858263 | 0.131825 |
| PC_40_5 | Phosphatidylcholine | 831128.8 | 201232.8 | 739081.1 | 228009.9 | 0.88925 | 0.138173 |
| SM_18_1_20_0 | Sphingomyelin | 7209489 | 2032720 | 6278504 | 1389395 | 0.870867 | 0.139589 |
| SM_d18_0_20_0 | Sphingomyelin | 4253927 | 1246744 | 3721001 | 1058382 | 0.874722 | 0.148823 |
| LPC_18_0 | Lysophosphatidylcholine | 2745444 | 1169095 | 2153218 | 526289.5 | 0.784288 | 0.156 |
| PC_34_0 | Phosphatidylcholine | 2739356 | 825261.9 | 2396250 | 719202.7 | 0.874749 | 0.158737 |
| PC_P_36_4_ | 1-O-alkyl-1'-enyl-phosphatidylcholine plasmalogen | 1296247 | 322751.5 | 1153659 | 242207.4 | 0.889999 | 0.165287 |
| PC_O_36_5_ | 1-O-alkyl-phosphatidylcholine plasmalogen | 1101481 | 294662.2 | 975060.5 | 220814.1 | 0.885227 | 0.185388 |
| PC_38_4 | Phosphatidylcholine | 7228902 | 2031120 | 6526654 | 1571204 | 0.902855 | 0.193966 |
| PC_P_38_5_ | 1-O-alkyl-1'-enyl-phosphatidylcholine plasmalogen | 677169.8 | 141204.4 | 620252.1 | 178435.7 | 0.915948 | 0.216434 |
| SM_18_1_18_1 | Sphingomyelin | 674367.3 | 142714.3 | 629351.5 | 173085.6 | 0.933247 | 0.217352 |
| SM_18_1_22_0 | Sphingomyelin | 4014475 | 1390994 | 3442530 | 1072589 | 0.857529 | 0.233789 |
| PC_36_2 | Phosphatidylcholine | 27021051 | 8902663 | 23418017 | 6696089 | 0.866658 | 0.234505 |
| SM_18_1_18_0 | Sphingomyelin | 1865238 | 540258.2 | 1674826 | 569242.2 | 0.897916 | 0.235415 |
| LPC_20_0 | Lysophosphatidylcholine | 24348.44 | 12158.75 | 18084.57 | 6747.877 | 0.742741 | 0.23641 |
| PC_38_6 | Phosphatidylcholine | 3521558 | 1101865 | 3216758 | 1141034 | 0.913447 | 0.242718 |
| SM_18_1_22_2 | Sphingomyelin | 1460987 | 414699.9 | 1355592 | 325710 | 0.92786 | 0.244203 |
| PC_30_0 | Phosphatidylcholine | 641899 | 188606.6 | 582866 | 174360 | 0.908034 | 0.249477 |
| PE_38_0 | Phosphatidylethanolamine | 4046.575 | 2394.196 | 3529.728 | 1488.055 | 0.872275 | 0.254648 |
| 9_HETE | HETE | 964.1463 | 772.2308 | 1331.429 | 703.6539 | 1.380941 | 0.260971 |
| PC_O_34_2_ | 1-O-alkyl-phosphatidylcholine plasmalogen | 1368364 | 350247.4 | 1175737 | 320191.9 | 0.859229 | 0.261676 |
| PE_38_3 | Phosphatidylethanolamine | 4169.184 | 2131.475 | 3573.267 | 1953.557 | 0.857066 | 0.269959 |
| PC_30_2 | Phosphatidylcholine | 733260.3 | 190270.3 | 649245.6 | 149927.6 | 0.885423 | 0.27134 |
| 12_13__EpOME | EpOME | 2293.626 | 1247.497 | 2985.877 | 1985.66 | 1.301815 | 0.276966 |
| PC_P_32_1_ | 1-O-alkyl-1'-enyl-phosphatidylcholine plasmalogen | 66115.84 | 16122.19 | 59310.5 | 14899.98 | 0.897069 | 0.280497 |
| PC_O_38_5_ | 1-O-alkyl-phosphatidylcholine plasmalogen | 1526168 | 366814.2 | 1357725 | 365024.2 | 0.88963 | 0.283709 |
| PC_36_1 | Phosphatidylcholine | 5201579 | 1954795 | 4534749 | 1965808 | 0.871802 | 0.290066 |
| 9_Nitrooleate | Nitro lipid | 68002.8 | 22673.12 | 85273.22 | 54548.66 | 1.253966 | 0.307237 |
| SM_18_1_20_2 | Sphingomyelin | 28902.23 | 10237.45 | 24813.66 | 6985.651 | 0.858538 | 0.309834 |
| 9_12_13_TriHOME | TriHOME | 1957.139 | 757.8002 | 2145.071 | 1176.441 | 1.096024 | 0.314999 |
| PC_O_32_1_ | 1-O-alkyl-phosphatidylcholine plasmalogen | 556653.3 | 152022.5 | 501724.6 | 134222.7 | 0.901323 | 0.322476 |
| 9_10_DiHOME | DiHOME | 4549.948 | 2982.262 | 5148.741 | 2237.186 | 1.131604 | 0.335718 |
| SM_18_1_16_1 | Sphingomyelin | 976271.7 | 286811.7 | 875626.2 | 189894.3 | 0.896908 | 0.336533 |
| LPC_16_0 | Lysophosphatidylcholine | 6073992 | 1996370 | 5446407 | 1672986 | 0.896677 | 0.342581 |
| PC_O_36_4_ | 1-O-alkyl-phosphatidylcholine plasmalogen | 1858059 | 455214.3 | 1670271 | 401624.1 | 0.898933 | 0.36779 |
| PC_P_36_2_ | 1-O-alkyl-1'-enyl-phosphatidylcholine plasmalogen | 814615.6 | 211201.8 | 700467.8 | 217376.7 | 0.859875 | 0.371014 |
| PE_P_34_2_ | 1-O-alkyl-1'-enyl-phosphatidylethanolamine plasmalogen | 2885.83 | 1508.708 | 3708.403 | 1800.944 | 1.285039 | 0.391315 |
| PC_P_36_3_ | 1-O-alkyl-1'-enyl-phosphatidylcholine plasmalogen | 1835845 | 450321 | 1654927 | 395454.7 | 0.901452 | 0.394338 |
| PC_40_6 | Phosphatidylcholine | 1535311 | 433157.5 | 1434140 | 601921.8 | 0.934104 | 0.400027 |
| PC_32_3 | Phosphatidylcholine | 31163.52 | 10892.04 | 27241.98 | 9295.263 | 0.874163 | 0.414004 |
| 5_HETE | HETE | 1375.631 | 994.1625 | 1790.94 | 967.3853 | 1.301904 | 0.414921 |
| PC_P_38_4_ | 1-O-alkyl-1'-enyl-phosphatidylcholine plasmalogen | 1501124 | 365307.7 | 1378399 | 371761.1 | 0.918245 | 0.41592 |
| LPC_14_0 | Lysophosphatidylcholine | 50587.67 | 29061.51 | 42077.47 | 23223.72 | 0.831773 | 0.416599 |
| PE_P_36_2_ | 1-O-alkyl-1'-enyl-phosphatidylethanolamine plasmalogen | 5272.518 | 3122.587 | 4531.091 | 1988.365 | 0.859379 | 0.423363 |
| Cer_32_1 | Ceramide | 21307.81 | 13583.42 | 14957.72 | 7159.948 | 0.701983 | 0.432138 |
| PC_O_36_3_ | 1-O-alkyl-phosphatidylcholine plasmalogen | 815752 | 213826.9 | 715118.8 | 206815.2 | 0.876638 | 0.435607 |
| 11_HETE | HETE | 1180.594 | 584.2973 | 1459.227 | 924.2434 | 1.236011 | 0.437831 |
| PC_P_36_1_ | 1-O-alkyl-1'-enyl-phosphatidylcholine plasmalogen | 1325360 | 580109.4 | 1193754 | 519038.7 | 0.900702 | 0.439479 |
| PE_34_2 | Phosphatidylethanolamine | 11539.41 | 4535.811 | 12443.16 | 8404.594 | 1.078319 | 0.444327 |
| PE_O_38_4_ | 1-O-alkyl-phosphatidylethanolamine plasmalogen | 4634.249 | 2126.197 | 4486.829 | 1982.748 | 0.968189 | 0.475591 |
| PC_36_5 | Phosphatidylcholine | 645012.7 | 300027.9 | 612357.8 | 249366.8 | 0.949373 | 0.485726 |
| LPE_18_0 | Lysophosphatidylethanolamine | 6785.931 | 3167.628 | 6409.246 | 3568.379 | 0.94449 | 0.493111 |
| SM_18_1_22_4 | Sphingomyelin | 6314.415 | 2733.656 | 5438.873 | 3812.578 | 0.861342 | 0.511375 |
| PC_40_0 | Phosphatidylcholine | 23893.25 | 15058 | 20223.08 | 17742.74 | 0.846393 | 0.516195 |
| 13_HODE | HODE | 3879.297 | 1870.561 | 3528.736 | 2050.861 | 0.909633 | 0.519741 |
| PE_38_4 | Phosphatidylethanolamine | 28111.73 | 13182.97 | 27957.97 | 15473.19 | 0.99453 | 0.529303 |
| PE_38_6 | Phosphatidylethanolamine | 9761.525 | 4869.904 | 11311.37 | 6153.849 | 1.158771 | 0.534496 |
| Cer_42_1 | Ceramide | 3967.493 | 2299.423 | 4447.665 | 1825.549 | 1.121026 | 0.539305 |
| PE_O_36_4_ | 1-O-alkyl-phosphatidylethanolamine plasmalogen | 6081.106 | 2979.442 | 6767.151 | 2529.393 | 1.112816 | 0.540674 |
| 4_HDoHE | HDoHE | 30056.84 | 27901.68 | 27124.05 | 29343.05 | 0.902425 | 0.541014 |
| PE_P_36_4_ | 1-O-alkyl-1'-enyl-phosphatidylethanolamine plasmalogen | 7456.504 | 3589.554 | 6999.462 | 2454.603 | 0.938706 | 0.561863 |
| PC_P_40_5_ | 1-O-alkyl-1'-enyl-phosphatidylcholine plasmalogen | 360439.6 | 87000.94 | 361023.6 | 124984.2 | 1.00162 | 0.574651 |
| PE_34_1 | Phosphatidylethanolamine | 7205.873 | 4630.368 | 6791.467 | 3582.931 | 0.94249 | 0.585583 |
| LacCer_d18_1_C16_0 | Lac-Ceramide | 5302.248 | 3264.066 | 3922.205 | 3146.969 | 0.739725 | 0.597318 |
| PC_34_5 | Phosphatidylcholine | 11971.12 | 4474.08 | 10987.23 | 5521.586 | 0.917811 | 0.606709 |
| LTB4 | Leukotriene | 1799.045 | 1378.33 | 2237.134 | 1835.437 | 1.243512 | 0.608291 |
| DHA__screen_ | Omega-3 fatty acid | 101091.4 | 49442.35 | 87677.91 | 44360.17 | 0.867313 | 0.626731 |
| PC_36_0 | Phosphatidylcholine | 282493.3 | 192539.6 | 322620.3 | 182154.9 | 1.142046 | 0.627232 |
| 9_HODE | HODE | 4712.3 | 3101.545 | 4458.458 | 2402.441 | 0.946132 | 0.645064 |
| 17_HDoHE | HDoHE | 12874.77 | 11956.76 | 14365.19 | 12387.01 | 1.115763 | 0.671699 |
| PE_P_36_3_ | 1-O-alkyl-1'-enyl-phosphatidylethanolamine plasmalogen | 6460.608 | 2986.789 | 6524.364 | 2462.43 | 1.009868 | 0.672609 |
| 19_20__EpDPE | EpDPE | 32331.07 | 29780.88 | 36289.31 | 30374.44 | 1.122428 | 0.675947 |
| PE_O_38_5_ | 1-O-alkyl-phosphatidylethanolamine plasmalogen | 10718.59 | 2972.169 | 11477.49 | 3714.663 | 1.070802 | 0.680621 |
| PE_36_2 | Phosphatidylethanolamine | 26601.08 | 14397.47 | 26636.19 | 14312.23 | 1.00132 | 0.69673 |
| PE_36_4 | Phosphatidylethanolamine | 11934.76 | 5014.519 | 13090.45 | 6925.793 | 1.096834 | 0.716814 |
| 14_HDoHE | HDoHE | 15553.09 | 15565.11 | 17220.76 | 15624.25 | 1.107225 | 0.740495 |
| 11_12__EpETrE | EpETrE | 1448.609 | 821.6042 | 1402.008 | 1004.748 | 0.96783 | 0.743097 |
| AA__screen_ | Omega-6 fatty acid | 63930.28 | 18103.86 | 74040.44 | 34078.42 | 1.158144 | 0.74331 |
| PC_O_40_6_ | 1-O-alkyl-phosphatidylcholine plasmalogen | 317989.8 | 84989.01 | 319214 | 110757.4 | 1.00385 | 0.750804 |
| PE_36_3 | Phosphatidylethanolamine | 8049.222 | 3804.784 | 8141.007 | 6103.43 | 1.011403 | 0.763589 |
| PC_P_30_0_ | 1-O-alkyl-1'-enyl-phosphatidylcholine plasmalogen | 194804.6 | 71196.48 | 200099.2 | 76720.36 | 1.027179 | 0.765536 |
| SM_18_1_18_2 | Sphingomyelin | 31445.92 | 10338.8 | 30912.22 | 10085.24 | 0.983028 | 0.789275 |
| LPC_20_5 | Lysophosphatidylcholine | 25406.16 | 21880.92 | 21668.53 | 12299.7 | 0.852885 | 0.802239 |
| Cer_34_1 | Ceramide | 15663.88 | 9865.222 | 13923.79 | 5478.577 | 0.888911 | 0.843956 |
| LPE_16_0 | Lysophosphatidylethanolamine | 6063.931 | 3053.175 | 6406.041 | 2401.631 | 1.056417 | 0.862267 |
| PE_P_38_5_ | 1-O-alkyl-1'-enyl-phosphatidylethanolamine plasmalogen | 8935.498 | 3627.366 | 8804.995 | 3554.277 | 0.985395 | 0.874219 |
| EPA__screen_ | Omega-3 fatty acid | 19807.37 | 15482.42 | 20538.29 | 14131.17 | 1.036901 | 0.879053 |
| TXB2 | Thromboxane | 2950.652 | 3917.369 | 5023.573 | 6314.871 | 1.70253 | 0.903512 |
| 9_10__EpOME | EpOME | 3194.002 | 1797.106 | 3194.245 | 1702.375 | 1.000076 | 0.951342 |
| PE_36_1 | Phosphatidylethanolamine | 4875.936 | 3254.78 | 5501.625 | 3339.914 | 1.128322 | 0.952202 |
| 6_trans_LTB4 | Leukotriene | 1717.096 | 1296.387 | 2114.933 | 2029.921 | 1.231692 | 0.960108 |
| PE_40_6 | Phosphatidylethanolamine | 5587.271 | 3515.204 | 5949.137 | 4677.158 | 1.064766 | 0.980391 |

Metabolomics data are in peak area

**Supplementary Table 2.** Group effect, time effect, and group-by-time interaction between all vitamin A groups. Metabolomics data are in peak area

|  |  |  |  |  |  |  |  |  | P-value | P-value | P-value |
| --- | --- | --- | --- | --- | --- | --- | --- | --- | --- | --- | --- |
|  |  |  |  |  |  |  |  |  |  |  |  |
| Metabolite Name | Mean(Adequate VA, Baseline) | Std Dev(Adequate VA, Baseline) | Mean(Adequate VA, Post-Supplement) | Std Dev(Adequate VA, Post-Supplement) | Mean(High VA, Baseline) | Std Dev(High VA, Baseline) | Mean(High VA, Post-Supplement) | Std Dev(High VA, Post-Supplement) | AA_Status_Group Effect | Time Effect | Group*Time Interaction |
| LPC_22_5 | 56452.74 | 30790.11 | 50415.97 | 24801.09 | 39371.7 | 22011.53 | 73078.22 | 33555.73 | 0.951094 | 0.042242 | 0.00217 |
| PE_38_0 | 4046.575 | 2394.196 | 3240.838 | 1699.311 | 3529.728 | 1488.055 | 5580.612 | 1416.162 | 0.30234 | 0.156514 | 0.002982 |
| LPC_22_4 | 18556.84 | 9612.048 | 15629.67 | 7946.31 | 11932.2 | 5009.154 | 23302.72 | 11324.77 | 0.856992 | 0.135956 | 0.003837 |
| LPC_20_3 | 203360.6 | 124803.6 | 157647.7 | 66817.47 | 119291.8 | 46751.66 | 211820.3 | 103056.7 | 0.603502 | 0.265002 | 0.008095 |
| LPC_20_4 | 466457.5 | 232583.8 | 401661.1 | 194279.8 | 347895 | 129521.3 | 563648.9 | 224698.2 | 0.907569 | 0.232156 | 0.009824 |
| PC_38_5 | 2613689 | 594295.4 | 2712513 | 857976 | 2314761 | 583957.7 | 3487533 | 989748.3 | 0.43445 | 0.00794 | 0.012571 |
| PC_34_4 | 63678.89 | 26731.81 | 57118.27 | 28349.29 | 45015.01 | 21207.8 | 74177.06 | 36708.38 | 0.690125 | 0.205652 | 0.016554 |
| PC_38_0 | 518675.2 | 172717.4 | 429535.7 | 192922.6 | 415042.4 | 124059 | 524260.9 | 145019.1 | 0.988855 | 0.812354 | 0.021786 |
| LPC_18_2 | 2522255 | 920851.2 | 2085065 | 688218.7 | 1951173 | 707727.3 | 3000127 | 1547834 | 0.762318 | 0.46637 | 0.022551 |
| PC_P_36_4_ | 1296247 | 322751.5 | 1272016 | 436822.4 | 1153659 | 242207.4 | 1566255 | 359255.8 | 0.440549 | 0.042038 | 0.022659 |
| 9_10__EpODE | 1179.861 | 515.6705 | 984.4557 | 638.1821 | 1116.433 | 935.3406 | 1646.087 | 1558.279 | 0.936552 | 0.46384 | 0.027281 |
| PC_38_4 | 7228902 | 2031120 | 7179523 | 2493298 | 6526654 | 1571204 | 9106957 | 2486113 | 0.337482 | 0.061608 | 0.028668 |
| PE_P_38_4_ | 13567.13 | 5357.209 | 9924.893 | 4081.865 | 10361.52 | 3701.125 | 11675.84 | 3668.043 | 0.666005 | 0.336659 | 0.030876 |
| PC_P_38_5_ | 677169.8 | 141204.4 | 669632.6 | 210939.5 | 620252.1 | 178435.7 | 817692.1 | 234828 | 0.87029 | 0.098686 | 0.033335 |
| PC_38_3 | 3751016 | 1419766 | 3612143 | 1464675 | 2916639 | 849155.5 | 4393636 | 1838421 | 0.769773 | 0.122111 | 0.037914 |
| PC_O_36_5_ | 1101481 | 294662.2 | 1100307 | 366028.3 | 975060.5 | 220814.1 | 1333793 | 335462.7 | 0.356018 | 0.040083 | 0.038801 |
| LPE_18_1 | 7018.156 | 3142.17 | 6152.817 | 3849.745 | 4802.661 | 4061.638 | 9698.595 | 8875.974 | 0.276115 | 0.278107 | 0.041346 |
| PC_O_38_5_ | 1526168 | 366814.2 | 1508477 | 557694.3 | 1357725 | 365024.2 | 1837396 | 453042.5 | 0.341647 | 0.058887 | 0.042339 |
| PC_O_34_3_ | 889361 | 262984.1 | 884251 | 413224.1 | 677252.7 | 219583.5 | 933603.5 | 300958.1 | 0.768532 | 0.159298 | 0.046361 |
| PE_P_38_5_ | 8935.498 | 3627.366 | 8371.518 | 4154.679 | 8804.995 | 3554.277 | 12453.99 | 5446.135 | 0.631861 | 0.321962 | 0.047734 |
| PE_38_3 | 4169.184 | 2131.475 | 3879.069 | 2000.231 | 3573.267 | 1953.557 | 6944.993 | 5674.899 | 0.518823 | 0.230446 | 0.053976 |
| PC_O_36_4_ | 1858059 | 455214.3 | 1815148 | 686042.5 | 1670271 | 401624.1 | 2142628 | 590026.6 | 0.223252 | 0.248843 | 0.054302 |
| PE_O_38_4_ | 4634.249 | 2126.197 | 3941.032 | 1815.238 | 4486.829 | 1982.748 | 6109.411 | 2615.067 | 0.187895 | 0.572642 | 0.054661 |
| SM_18_1_22_2 | 1460987 | 414699.9 | 1456188 | 450102.6 | 1355592 | 325710 | 1901957 | 615427.3 | 0.294986 | 0.070204 | 0.055687 |
| LPC_16_1 | 141041 | 44423.52 | 133699.4 | 49406.61 | 93036.84 | 41603.5 | 142444.2 | 77980.67 | 0.09727 | 0.205025 | 0.060546 |
| PC_O_40_0_ | 146140.6 | 33308.01 | 147236.7 | 45405.92 | 117797.9 | 36076.77 | 163394.9 | 61983.7 | 0.637237 | 0.051228 | 0.062181 |
| PC_36_3 | 8549163 | 2628956 | 7995019 | 2228722 | 6980137 | 1562662 | 9550203 | 3527315 | 0.883789 | 0.26296 | 0.062226 |
| SM_18_1_20_1 | 616154.1 | 142513.1 | 609673.6 | 168689.5 | 510611.6 | 127111 | 665952.8 | 211375 | 0.451122 | 0.120075 | 0.063322 |
| PC_P_32_1_ | 66115.84 | 16122.19 | 68759.85 | 17079.75 | 59310.5 | 14899.98 | 78155.3 | 20583.39 | 0.685161 | 0.016551 | 0.063834 |
| PC_P_38_4_ | 1501124 | 365307.7 | 1522361 | 575700.8 | 1378399 | 371761.1 | 1857756 | 447571.3 | 0.276558 | 0.043817 | 0.064473 |
| PC_40_0 | 23893.25 | 15058 | 22109.33 | 11518.24 | 20223.08 | 17742.74 | 40655.3 | 32793.39 | 0.487808 | 0.123575 | 0.067057 |
| PC_36_4 | 9867568 | 2525176 | 9676187 | 2583038 | 9083099 | 1947708 | 11945745 | 3412769 | 0.483825 | 0.121573 | 0.067134 |
| LPC_18_1 | 1531989 | 726156.8 | 1394636 | 478753.7 | 1140961 | 465006.8 | 1658987 | 737429.1 | 0.695616 | 0.233094 | 0.067283 |
| PC_34_3 | 904774.4 | 206708.2 | 868784 | 286270.6 | 669745.2 | 220625.2 | 933844.2 | 432799.5 | 0.109684 | 0.275885 | 0.070303 |
| PC_P_36_2_ | 814615.6 | 211201.8 | 825548.9 | 343908.5 | 700467.8 | 217376.7 | 947614.9 | 383035.9 | 0.545506 | 0.163577 | 0.070891 |
| PC_40_5 | 831128.8 | 201232.8 | 900961.7 | 363560.2 | 739081.1 | 228009.9 | 1037455 | 321403 | 0.911347 | 0.037983 | 0.077593 |
| PC_P_36_3_ | 1835845 | 450321 | 1825778 | 677238 | 1654927 | 395454.7 | 2113218 | 602617.7 | 0.29512 | 0.217293 | 0.079208 |
| PC_O_36_3_ | 815752 | 213826.9 | 823865.3 | 316728 | 715118.8 | 206815.2 | 951851.2 | 352842.6 | 0.552206 | 0.138274 | 0.08647 |
| LPC_22_6 | 155645.6 | 98786.72 | 148520.9 | 72341.04 | 107962.8 | 54339.55 | 175660.7 | 99460.51 | 0.338856 | 0.142866 | 0.088386 |
| PE_O_38_5_ | 10718.59 | 2972.169 | 10171.13 | 4772.673 | 11477.49 | 3714.663 | 16779.27 | 10548.38 | 0.485042 | 0.494256 | 0.0885 |
| 20_HETE | 5282.629 | 3018.758 | 11847.28 | 14050.65 | 15432.72 | 25724.72 | 5618.55 | 4410.184 | 0.669699 | 0.889355 | 0.089193 |
| PC_34_5 | 11971.12 | 4474.08 | 10479.99 | 4875.027 | 10987.23 | 5521.586 | 14931.65 | 8175.795 | 0.783808 | 0.587029 | 0.090381 |
| PC_O_34_2_ | 1368364 | 350247.4 | 1351238 | 550252.9 | 1175737 | 320191.9 | 1533156 | 629944.6 | 0.709634 | 0.255405 | 0.090595 |
| PC_P_40_5_ | 360439.6 | 87000.94 | 382101.9 | 151966.7 | 361023.6 | 124984.2 | 483388.6 | 159457.2 | 0.392185 | 0.060203 | 0.095452 |
| PC_40_7 | 141967.1 | 40582.99 | 144366.9 | 48251.68 | 113682.8 | 40709.93 | 157567.1 | 55304.51 | 0.494996 | 0.065942 | 0.097699 |
| PC_30_2 | 733260.3 | 190270.3 | 722455.9 | 207800.1 | 649245.6 | 149927.6 | 825934.1 | 246562.6 | 0.607494 | 0.144164 | 0.098008 |
| PE_36_1 | 4875.936 | 3254.78 | 4494.914 | 3768.807 | 5501.625 | 3339.914 | 10031.94 | 9048.729 | 0.122342 | 0.499957 | 0.100634 |
| LPC_18_0 | 2745444 | 1169095 | 2517761 | 907449.4 | 2153218 | 526289.5 | 2975323 | 1138177 | 0.949507 | 0.301247 | 0.100831 |
| PE_P_36_2_ | 5272.518 | 3122.587 | 3824.855 | 1784.431 | 4531.091 | 1988.365 | 7723.367 | 11272.57 | 0.499811 | 0.906135 | 0.102733 |
| 12_13_DiHOME | 1249.78 | 909.7836 | 1003.077 | 648.9365 | 1128.972 | 1086.195 | 1744.732 | 1374.711 | 0.644303 | 0.562611 | 0.10436 |
| SM_18_1_22_0 | 4014475 | 1390994 | 3881735 | 1242236 | 3442530 | 1072589 | 4653722 | 1801519 | 0.640667 | 0.197151 | 0.104879 |
| SM_18_1_20_0 | 7209489 | 2032720 | 6775811 | 1593311 | 6278504 | 1389395 | 7954100 | 2838436 | 0.792671 | 0.343421 | 0.105636 |
| Protectin_DX | 9854.093 | 5235.219 | 15972.73 | 11162.76 | 22455.42 | 29609.26 | 13295.85 | 9403.082 | 0.770986 | 0.758087 | 0.108063 |
| PC_36_5 | 645012.7 | 300027.9 | 705494.2 | 338739.8 | 612357.8 | 249366.8 | 1356157 | 1407504 | 0.638102 | 0.034411 | 0.108526 |
| PC_38_6 | 3521558 | 1101865 | 3675363 | 1208893 | 3216758 | 1141034 | 4341871 | 1333074 | 0.77689 | 0.060052 | 0.110289 |
| SM_18_1_16_0 | 10283404 | 2737443 | 9844598 | 2911281 | 8906080 | 1865549 | 11354889 | 4941483 | 0.784367 | 0.39172 | 0.115651 |
| PC_36_2 | 27021051 | 8902663 | 26074616 | 7971566 | 23418017 | 6696089 | 30977011 | 12160441 | 0.607543 | 0.228935 | 0.118589 |
| SM_18_1_16_1 | 976271.7 | 286811.7 | 950352.2 | 252436.2 | 875626.2 | 189894.3 | 1094468 | 307124 | 0.531442 | 0.223871 | 0.120784 |
| PE_36_3 | 8049.222 | 3804.784 | 7089.343 | 3974.665 | 8141.007 | 6103.43 | 11681.23 | 9402.194 | 0.937873 | 0.494288 | 0.125688 |
| Cer_32_1 | 21307.81 | 13583.42 | 20256.71 | 7391.913 | 14957.72 | 7159.948 | 23021.47 | 8663.966 | 0.748316 | 0.043601 | 0.125807 |
| PC_O_32_1_ | 556653.3 | 152022.5 | 561873.6 | 186174.4 | 501724.6 | 134222.7 | 640123.7 | 170991.2 | 0.741275 | 0.102858 | 0.129178 |
| PC_34_2 | 37984628 | 10983284 | 35837933 | 7743268 | 33106802 | 7486919 | 41003362 | 13794982 | 0.805286 | 0.349053 | 0.129288 |
| PC_34_1 | 30386412 | 8215054 | 30152602 | 8281181 | 26357821 | 6887338 | 33550334 | 10654778 | 0.978117 | 0.187794 | 0.133635 |
| LPC_16_0 | 6073992 | 1996370 | 5822830 | 1900939 | 5446407 | 1672986 | 6931881 | 2058621 | 0.778838 | 0.314014 | 0.1347 |
| PE_36_2 | 26601.08 | 14397.47 | 24658.76 | 13020.22 | 26636.19 | 14312.23 | 43656.94 | 30354.66 | 0.606842 | 0.25849 | 0.141835 |
| LTB5 | 1426.138 | 483.045 | 2306.142 | 1329.586 | 1927.349 | 965.8169 | 1903.988 | 1313.242 | 0.623323 | 0.546939 | 0.150852 |
| LPC_20_0 | 24348.44 | 12158.75 | 23191.41 | 12084.2 | 18084.57 | 6747.877 | 25877.25 | 13660.15 | 0.887722 | 0.386102 | 0.156484 |
| PE_P_36_4_ | 7456.504 | 3589.554 | 7506.511 | 4489.589 | 6999.462 | 2454.603 | 9813.331 | 4983.095 | 0.410593 | 0.613887 | 0.163588 |
| PC_O_40_6_ | 317989.8 | 84989.01 | 357257.4 | 142063.1 | 319214 | 110757.4 | 423947.1 | 109244.1 | 0.355888 | 0.031369 | 0.171892 |
| PC_P_36_1_ | 1325360 | 580109.4 | 1358343 | 559792.9 | 1193754 | 519038.7 | 1691797 | 708672.5 | 0.771056 | 0.092362 | 0.173065 |
| PC_30_1 | 6820271 | 2014381 | 6687342 | 2081271 | 5765449 | 1265298 | 7109874 | 2124459 | 0.634885 | 0.268266 | 0.174157 |
| PE_38_4 | 28111.73 | 13182.97 | 26770.84 | 11072.36 | 27957.97 | 15473.19 | 40736.62 | 25540.45 | 0.535799 | 0.286114 | 0.175281 |
| PC_36_1 | 5201579 | 1954795 | 5282277 | 2309440 | 4534749 | 1965808 | 6256439 | 3032467 | 0.790187 | 0.207529 | 0.177034 |
| PC_34_0 | 2739356 | 825261.9 | 2759519 | 837972.5 | 2396250 | 719202.7 | 3024256 | 978079.2 | 0.999051 | 0.189011 | 0.184837 |
| PC_P_34_2_ | 1123057 | 297505.6 | 1119565 | 438645.1 | 872541.8 | 260161 | 1112008 | 342995.2 | 0.428079 | 0.198657 | 0.185762 |
| SM_d18_0_20_0 | 4253927 | 1246744 | 4238543 | 1210664 | 3721001 | 1058382 | 4634417 | 1491816 | 0.943188 | 0.241032 | 0.197308 |
| PE_P_36_3_ | 6460.608 | 2986.789 | 5972.12 | 2673.47 | 6524.364 | 2462.43 | 8570.644 | 4499.172 | 0.268325 | 0.576396 | 0.200784 |
| SM_18_1_20_2 | 28902.23 | 10237.45 | 26651.29 | 9146.369 | 24813.66 | 6985.651 | 30669.32 | 13200.76 | 0.993008 | 0.702864 | 0.20157 |
| PC_40_6 | 1535311 | 433157.5 | 1694550 | 660231.9 | 1434140 | 601921.8 | 1856917 | 641091.6 | 0.847083 | 0.073187 | 0.202313 |
| LPC_20_5 | 25406.16 | 21880.92 | 31620.88 | 22880.56 | 21668.53 | 12299.7 | 64284.23 | 73269.35 | 0.452462 | 0.00915 | 0.202491 |
| PC_32_0 | 2246300 | 561407 | 2265642 | 609774.6 | 1937715 | 466596.3 | 2408079 | 748022.3 | 0.649602 | 0.209345 | 0.202662 |
| LPE_18_0 | 6785.931 | 3167.628 | 6494.871 | 3372.17 | 6409.246 | 3568.379 | 9850.079 | 6217.641 | 0.590685 | 0.369615 | 0.211544 |
| 11_12__EpETrE | 1448.609 | 821.6042 | 816.079 | 691.4682 | 1628.843 | 1307.372 | 975.4419 | 690.5007 | 0.930382 | 0.046371 | 0.213686 |
| LacCer_d18_1_C16_0 | 5302.248 | 3264.066 | 5590.887 | 3635.388 | 3922.205 | 3146.969 | 8614.101 | 14065.48 | 0.995233 | 0.144609 | 0.219406 |
| TXB2 | 2950.652 | 3917.369 | 828.6441 | 1015.519 | 5848.629 | 6873.134 | 2018.941 | 1709.408 | 0.177353 | 0.05339 | 0.256373 |
| SM_18_1_18_0 | 1865238 | 540258.2 | 1895520 | 807341.3 | 1674826 | 569242.2 | 1976393 | 579308.5 | 0.912739 | 0.472126 | 0.263587 |
| PC_32_2 | 689729.4 | 182086.8 | 708541.3 | 202277.1 | 591968.9 | 151183.1 | 701286.8 | 169838 | 0.655205 | 0.176966 | 0.26982 |
| 11_HETE | 1180.594 | 584.2973 | 759.1424 | 673.4183 | 1666.443 | 1198.869 | 996.034 | 785.6958 | 0.267588 | 0.021934 | 0.281009 |
| PE_34_2 | 11539.41 | 4535.811 | 11183.93 | 6591.675 | 12443.16 | 8404.594 | 18263.68 | 15456.37 | 0.774565 | 0.586743 | 0.281476 |
| 9_Nitrooleate | 68002.8 | 22673.12 | 81810.02 | 39904.9 | 107808.1 | 101883.9 | 73970.16 | 47835.96 | 0.81603 | 0.837288 | 0.287508 |
| PE_36_4 | 11934.76 | 5014.519 | 13203.57 | 8710.312 | 13090.45 | 6925.793 | 19853.94 | 15048.6 | 0.532234 | 0.219979 | 0.293619 |
| SM_18_1_18_2 | 31445.92 | 10338.8 | 31016.58 | 8805.473 | 30912.22 | 10085.24 | 35832.56 | 10230.66 | 0.634751 | 0.388361 | 0.306143 |
| SM_d18_0_16_0 | 991125.3 | 273337.8 | 1018289 | 332546.9 | 868798.1 | 177631.4 | 1078362 | 405593.8 | 0.531547 | 0.283907 | 0.311252 |
| PC_32_1 | 3042833 | 800294.6 | 3136296 | 1100370 | 2466962 | 628328.1 | 3019560 | 1068431 | 0.26935 | 0.337163 | 0.330121 |
| 19_20__EpDPE | 32331.07 | 29780.88 | 32957.04 | 31064.5 | 35419.78 | 29462.65 | 31585.84 | 19292.52 | 0.254621 | 0.833259 | 0.331638 |
| Resolvin_D2 | 6501.571 | 4898.782 | 4485.522 | 5279.608 | 3446.877 | 2332.315 | 3212.943 | 2782.722 | 0.263056 | 0.100107 | 0.351175 |
| SM_18_1_18_1 | 674367.3 | 142714.3 | 679825.6 | 207516.8 | 629351.5 | 173085.6 | 723907.3 | 169329.1 | 0.925512 | 0.305518 | 0.364019 |
| ALA__screen_ | 4772.035 | 3188.372 | 3681.434 | 4386.668 | 2733.927 | 1973.594 | 3000.714 | 2461.294 | 0.159834 | 0.326976 | 0.386663 |
| PE_O_36_4_ | 6081.106 | 2979.442 | 6477.195 | 3149.544 | 6767.151 | 2529.393 | 9584.441 | 8181.789 | 0.230648 | 0.317435 | 0.393535 |
| PE_40_6 | 5587.271 | 3515.204 | 5942.997 | 3173.684 | 5949.137 | 4677.158 | 7813.372 | 5848.688 | 0.521915 | 0.172228 | 0.399586 |
| 14_HDoHE | 15553.09 | 15565.11 | 15843.52 | 14983.78 | 16922.94 | 15100.03 | 15528.45 | 10241.9 | 0.291922 | 0.890659 | 0.404017 |
| SM_18_1_22_4 | 6314.415 | 2733.656 | 7691.534 | 4935.656 | 5438.873 | 3812.578 | 7559.754 | 4986.679 | 0.611686 | 0.292495 | 0.40949 |
| DHA__screen_ | 101091.4 | 49442.35 | 91629.45 | 49230.19 | 92793.96 | 47115.55 | 97820.75 | 55278.56 | 0.89223 | 0.957668 | 0.418449 |
| LPE_18_2 | 10340.07 | 5778.329 | 9085.98 | 4513.11 | 7955.349 | 3721.424 | 12414.89 | 8901.575 | 0.825129 | 0.68664 | 0.457489 |
| SM_d18_0_18_0 | 414170.9 | 121661.3 | 441970.2 | 168691.2 | 348043 | 107417.6 | 423179.1 | 153194.2 | 0.255667 | 0.279757 | 0.474879 |
| 17_HDoHE | 12874.77 | 11956.76 | 15573.17 | 15056.96 | 14170.32 | 11960.26 | 14048.47 | 9854.326 | 0.64401 | 0.821618 | 0.496892 |
| 9_10__EpOME | 3194.002 | 1797.106 | 2063.053 | 2658.378 | 3610.644 | 2300.412 | 2745.882 | 3189.223 | 0.848761 | 0.01439 | 0.510239 |
| 9_HETE | 964.1463 | 772.2308 | 511.3951 | 405.0994 | 1510.974 | 971.2394 | 579.0372 | 484.1376 | 0.403136 | 0.002554 | 0.515352 |
| 9_12_13_TriHOME | 1957.139 | 757.8002 | 2496.387 | 1284.49 | 2517.545 | 1834.722 | 2338.828 | 1158.083 | 0.990941 | 0.44505 | 0.51954 |
| PE_34_1 | 7205.873 | 4630.368 | 7329.9 | 3788.529 | 6791.467 | 3582.931 | 9492.611 | 6142.648 | 0.839668 | 0.37058 | 0.523446 |
| PC_P_30_0_ | 194804.6 | 71196.48 | 198037.5 | 71874.86 | 200099.2 | 76720.36 | 230730.5 | 75075.21 | 0.327912 | 0.434757 | 0.534865 |
| LPC_14_0 | 50587.67 | 29061.51 | 60622.18 | 38214.65 | 42077.47 | 23223.72 | 58844.94 | 26401.21 | 0.580784 | 0.114731 | 0.566877 |
| PE_P_34_2_ | 2885.83 | 1508.708 | 4236.585 | 2228.915 | 3708.403 | 1800.944 | 6068.98 | 8668.09 | 0.63977 | 0.2106 | 0.578868 |
| 12_13__EpOME | 2293.626 | 1247.497 | 2371.877 | 1922.735 | 3374.625 | 2434.767 | 3089.832 | 2714.873 | 0.822599 | 0.728893 | 0.615597 |
| Cer_34_1 | 15663.88 | 9865.222 | 16301.59 | 4895.063 | 13923.79 | 5478.577 | 14750.57 | 6393.656 | 0.632064 | 0.367536 | 0.639724 |
| PC_32_3 | 31163.52 | 10892.04 | 35317.9 | 13573.41 | 27241.98 | 9295.263 | 34358.62 | 12696.39 | 0.590238 | 0.082082 | 0.658145 |
| 6_trans_LTB4 | 1717.096 | 1296.387 | 1023.088 | 1241.745 | 2486.507 | 2428.43 | 1209.697 | 1210.638 | 0.901457 | 0.02882 | 0.700618 |
| 10_Nitrooleate | 1496.608 | 1102.419 | 1008.374 | 601.0002 | 1035.744 | 655.3355 | 889.5525 | 579.2257 | 0.337415 | 0.142935 | 0.709247 |
| PC_30_0 | 641899 | 188606.6 | 665582.6 | 212893.5 | 582866 | 174360 | 638782 | 194642.8 | 0.471779 | 0.533146 | 0.711402 |
| EPA__screen_ | 19807.37 | 15482.42 | 20854.36 | 10965.04 | 24170.5 | 19578.57 | 24222.19 | 28894.83 | 0.642658 | 0.720046 | 0.723805 |
| Cer_42_1 | 3967.493 | 2299.423 | 3859.577 | 2764.413 | 4447.665 | 1825.549 | 4917.07 | 5445.204 | 0.784443 | 0.532491 | 0.738132 |
| AA__screen_ | 63930.28 | 18103.86 | 49339.88 | 26360.82 | 84108.89 | 50980.29 | 55845.21 | 24837.57 | 0.524841 | 0.023396 | 0.748284 |
| 9_10_DiHOME | 4549.948 | 2982.262 | 4564.894 | 1762.899 | 5046.728 | 2191.712 | 6524.275 | 2603.107 | 0.025031 | 0.141595 | 0.770042 |
| 13_HODE | 3879.297 | 1870.561 | 3244.352 | 2219.139 | 4255.117 | 3438.027 | 3996.536 | 3985.493 | 0.761755 | 0.29058 | 0.778355 |
| 5_HETE | 1375.631 | 994.1625 | 1198.04 | 1161.66 | 2436.822 | 2669.539 | 1435.32 | 1240.605 | 0.259609 | 0.090865 | 0.808372 |
| 12_HETE | 4610.538 | 6690.156 | 3580.884 | 7806.454 | 13489.56 | 16555.58 | 3965.464 | 2315.637 | 0.023697 | 0.011885 | 0.851122 |
| 4_HDoHE | 30056.84 | 27901.68 | 31339.51 | 29910.28 | 27136.9 | 28275.72 | 24849.76 | 23765.66 | 0.389068 | 0.637881 | 0.86388 |
| LTB4 | 1799.045 | 1378.33 | 1509.03 | 1866.218 | 2990.558 | 3412.173 | 1161.229 | 1046.543 | 0.704698 | 0.0457 | 0.867384 |
| 9_HODE | 4712.3 | 3101.545 | 3784.089 | 3860.459 | 4958.68 | 3018.738 | 3621.005 | 3418.036 | 0.889185 | 0.066824 | 0.915105 |
| LPE_16_0 | 6063.931 | 3053.175 | 6244.506 | 3832.099 | 6406.041 | 2401.631 | 7387.645 | 4574.164 | 0.435751 | 0.781147 | 0.966634 |
| PE_38_6 | 9761.525 | 4869.904 | 11953.22 | 5115.404 | 11311.37 | 6153.849 | 14590.49 | 10437.67 | 0.366321 | 0.072904 | 0.996126 |
| PC_36_0 | 282493.3 | 192539.6 | 156266.5 | 144074.3 | 322620.3 | 182154.9 | 221611.2 | 235666.6 | 0.769441 | 0.011846 | 0.998787 |

**Supplementary Table 3** – ChemRICH chemical similarity enrichment analysis comparing the adequate vitamin A versus the high vitamin A group at baseline

| **Cluster name** | **Cluster size** | **Key compound** | **Altered metabolites** | **Increased** | **Decreased** | **Increased ratio** | **Altered Ratio** | **p-values** | **FDR** |
| --- | --- | --- | --- | --- | --- | --- | --- | --- | --- |
| Unsaturated_phosphatidylcholines | 36 | PC 34:3 | 3 | 0 | 3 | 0 | 0.08 | 0.000000023 | 0.00000038 |
| Unsaturated_lysophosphatidylcholines | 9 | LPC 16:1 | 6 | 0 | 6 | 0 | 0.7 | 0.00000013 | 0.000001 |
| ceramides | 5 | SM 18:1 22:0 | 0 | 0 | 0 |  | 0 | 1 | 1 |
| DiHETE | 3 | LTB5 | 0 | 0 | 0 |  | 0 | 1 | 1 |
| DiHOME | 3 | 12,13-DiHOME | 0 | 0 | 0 |  | 0 | 1 | 1 |
| EpETrE | 3 | 9(10)-EpODE | 0 | 0 | 0 |  | 0 | 1 | 1 |
| EpODE | 3 | 19(20)-EpDPE | 0 | 0 | 0 |  | 0 | 1 | 1 |
| HETE | 6 | 12-HETE | 0 | 0 | 0 |  | 0 | 1 | 1 |
| OH-FA_22_6_1 | 4 | Resolvin D2 | 0 | 0 | 0 |  | 0 | 1 | 1 |
| phosphatidylethanolamines | 20 | LPE 18:1 | 1 | 0 | 1 | 0 | 0.05 | 1 | 1 |
| phospholipid ethers | 6 | PC (O40:0) | 1 | 0 | 1 | 0 | 0.2 | 1 | 1 |
| Saturated_lysophosphatidylcholines | 5 | LPC 18:0 | 0 | 0 | 0 |  | 0 | 1 | 1 |
| Saturated_phosphatidylcholines | 7 | PC 32:0 | 0 | 0 | 0 |  | 0 | 1 | 1 |
| Saturated_sphingomyelins | 3 | SM d18:0 16:0 | 0 | 0 | 0 |  | 0 | 1 | 1 |
| Unsaturated FA | 4 | Alpha-Linolenic Acid | 1 | 0 | 1 | 0 | 0.2 | 1 | 1 |
| Unsaturated_sphingomyelins | 7 | SM 18:1 20:1 | 1 | 0 | 1 | 0 | 0.1 | 1 | 1 |

**Supplementary Figure 1:** Principal component analysis discriminating adequate and high vitamin A groups at baseline and after intervention.


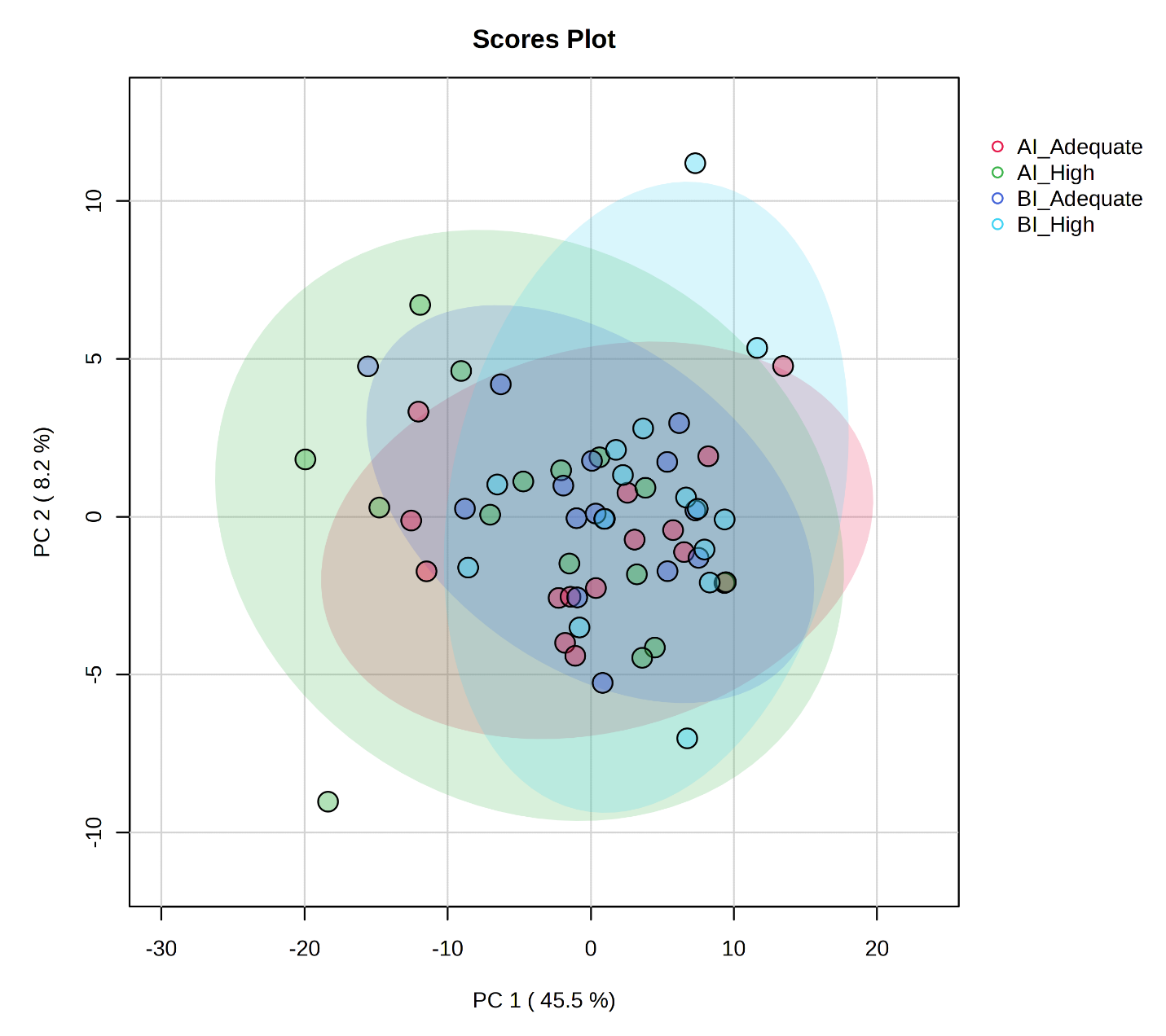


**Supplementary Figure 2:** Partial least squares discriminant analysis discriminating adequate and high vitamin A groups at baseline and after intervention.


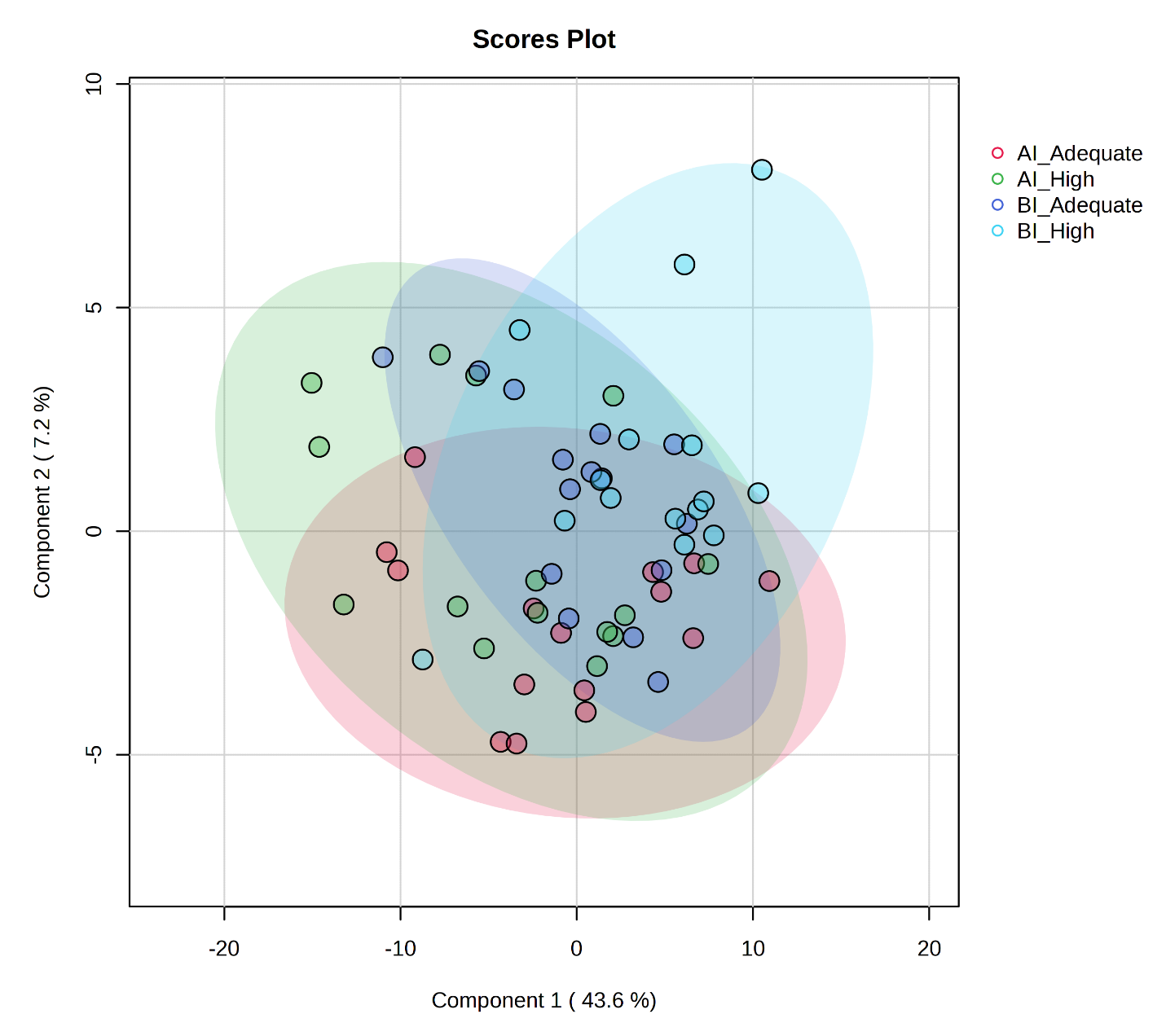

Supplement: Cohen et al. supplementary material [file S0007114525103656sup001.docx]
